# Patterns and Processes of Genomic Evolution Inferred From the Ten Smallest Vertebrate Genomes

**DOI:** 10.1002/advs.202417251

**Published:** 2025-09-10

**Authors:** Kaiqiang Liu, Qian Wang, Ning Wang, Lingfeng Meng, Shuo Li, Hong‐Yan Wang, Yuyan Liu, Qian Liu, Yangqing Zhang, Lucas B. Doretto, Mengqi Zhang, Yating Qin, Shanshan Pan, Shenglei Han, Weijing Li, Shanshan Liu, Fengtao Gao, Axel Meyer, Jussi Taipale, Guangyi Fan, Manfred Schartl, Jilin Zhang, Changwei Shao

**Affiliations:** ^1^ State Key Laboratory of Mariculture Biobreeding and Sustainable Goods Yellow Sea Fisheries Research Institute Chinese Academy of Fishery Sciences Qingdao Shandong 266071 China; ^2^ Laboratory for Marine Fisheries Science and Food Production Processes Qingdao Marine Science and Technology Center Qingdao Shandong 266237 China; ^3^ Department of Biophysics and Biochemistry Karolinska Institute Stockholm 17177 Sweden; ^4^ BGI‐Qingdao BGI‐Shenzhen Qingdao Shandong 266555 China; ^5^ BGI‐Shenzhen Shenzhen Guangdong 518083 China; ^6^ Weifang Municipal Key Laboratory of Fishery Resources Development & Comprehensive Utilization College of Biology and Oceanography Weifang University Weifang 261053 China; ^7^ Department of Biology University of Konstanz 78457 Konstanz Germany; ^8^ CAS Key Laboratory of Tropical Marine Bio‐Resources and Ecology South China Sea Institute of Oceanology Chinese Academy of Sciences Guangzhou 510301 China; ^9^ Museum of Comparative Zoology Harvard University Cambridge MA 02138 USA; ^10^ Department of Biochemistry University of Cambridge Cambridge CB2 1QW U.K; ^11^ Developmental Biochemistry Biozentrum University of Würzburg 97074 Würzburg Germany; ^12^ Tung Biomedical Sciences Centre City University of Hong Kong Hong Kong S.A.R 999077 China; ^13^ Department of Precision Diagnostic and Therapeutic Technology The City University of Hong Kong Shenzhen Futian Research Institute Shenzhen 518053 China; ^14^ Department of Biomedical Sciences City University of Hong Kong Hong Kong S.A.R 999077 China

**Keywords:** genome evolution, most compact genome, pufferfish genome

## Abstract

Pufferfish exhibit the smallest vertebrate genomes, making them ideal models for investigating evolutionary patterns and processes that affect genome size. While the *Takifugu rubripes* genome was fully sequenced two decades ago, key evolutionary drivers remain elusive. We sequenced 10 pufferfish genomes and generated 35 transcriptomes and 13 methylomes to understand genomic evolutionary mechanisms. Comparative genomics revealed that transposable element suppression—rather than lineage‐specific conserved element loss—primarily underlies genome compaction. This is mediated by reductions in transposon‐associated enzymes that limit transposable element propagation and modify DNA repair mechanisms that promote genomic streamlining. Based on resolved phylogeny among nine *Takifugu*, it is found that introgression drives speciation of *T. niphobles* and *T. oblongus*, while long‐term linked selection dominates divergence in other species. Positive selection analyses highlighted mechanotransduction pathway genes (integrins, ion channel transport) that are functionally convergent with mammalian lung cell mechanisms, potentially supporting inflation‐based anti‐predatory strategies. Additionally, positive selection variants in genes that control lineage‐specific skin patterning and coloration, which are either selected for or are rewired in regulatory processes, might suggest a role for pigmentation during the rapid speciation of this lineage. This findings shed light on mechanisms enabling extreme vertebrate genome compaction and provide insights for genome engineering applications.

## Graphical abstract

By integrating phylogenetic relationships derived from nuclear DNA with population genetic analyses across ten pufferfish species, the genetic differentiation mechanisms underpinning the radiative speciation of the most compact vertebrate genomes has elucidated. Furthermore, genomic loci associated with phenotypic innovations is identified, driven by variations in chromatin organization and the modulation of non‐coding regulatory elements.

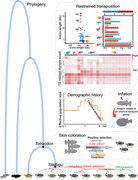

## 1 Introduction

In the foreword of *On the Origin of Species*, Darwin described speciation as the “mystery of mysteries”. Despite over 150 years of research, the mechanisms driving this fundamental evolutionary process remain elusive, leaving key questions about the origin of biodiversity unresolved.^[^ ^1^ ^]^ Decades of studies across various lineages in the tree of life have provided valuable molecular insight into genetic differentiation.^[^ ^2^ ^]^ However, speciation remains under intense debate due to discrete or static sampling from diverged species and the complexity of large genomes^3^. Consequently, lineages with compact genomes, such as those found among pufferfish, might provide powerful systems to better understand both the evolution of compact genomes as well as the genomic differences that distinguish species from each other.

The pufferfish genus *Takifugu* is one such model.^[^ ^3^ ^]^ These fish have the most compact genomes among vertebrates,^[^ ^4^ ^]^ and the roughly 25 species in the genus have diverged within a remarkably short evolutionary time (1 to 5 million years).^[^ ^5^ ^]^ Notably, this rapid radiation occurred without significant genome size variation, preserving a genomic architecture ideal for evolutionary inference. The sequencing of *Takifugu rubripes*,the first teleost fish genome to be sequenced,^[^ ^4^ ^]^ revealed a ≈10‐fold reduction in genome size compared to humans and yet maintained conserved synteny and orthology for ≈70% of human genes.^[^ ^4^ ^]^ Beyond its genomic minimalism, *Takifugu* offers unique insights into adaptive evolution. Traits such as rapid body inflation and tetrodotoxin resistance,^[^ ^6^, ^7^, ^8^ ^]^ which help deter predators and provide other survival advantages, highlight the interplay between genomic architecture and ecological adaptation. These features make the *Takifugu* lineage an interesting system for probing both vertebrate genome evolution and phenotypic innovation under constrained genomic regimes.

Previous efforts to reconstruct the phylogeny of *Takifugu* relied on mitochondrial genomes, which inherently reflects only maternal evolutionary histories. This approach has yielded incomplete and conflicting phylogenetic relationships across the genus.^[^ ^5^ ^]^ While early comparative genomic studies understandably had to be based on incomplete genome assemblies (e.g., *T. rubripes, T. niphobles, T. bimaculatus, T. obscurus*, and *T. flavidus*)^[^ ^9^, ^10^, ^11^, ^12^ ^]^ and identified minimal large‐scale genomic rearrangements,^[^ ^13^ ^]^ they revealed a striking paradox: rapid morphological diversification appears to be linked to only subtle divergence in protein‐coding regions.^[^ ^14^ ^]^ Here, we set out to obtain more complete taxon sampling and chromosome‐level genomic information to study genome evolution and adaptive diversification^[^ ^10^, ^11^, ^12^ ^]^ of traits such as body inflation and skin pattern.

The advent of high‐throughput sequencing and computational genomics tools has revolutionized the ability to dissect vertebrate genome evolution. Such tools have confirmed, for example, that functional elements are physically organized and dynamically orchestrated. Recent studies have uncovered how dynamic processes—such as the birth of genes, regulatory turnover, and non‐coding sequence evolution—sculpt morphological and physiological diversity.^[^ ^15^, ^16^, ^17^, ^18^ ^]^ However, the ways in which evolution impacts genetic differentiation remain incompletely understood. Studies on the explosive radiation of cichlid fishes in the three great East African Lakes—Victoria, Malawi, and Tanganyika^[^ ^19^, ^20^, ^21^, ^22^, ^23^, ^24^ ^]^ have shown that distinct morphological traits are associated with diversified genomic elements.^[^ ^25^, ^26^ ^]^ Similarly, *Takifugu* with their conserved genome size yet remarkable phenotypic divergence, offer a complementary marine model to unravel how genomic constraint and innovation coexist during speciation, a topic that is notoriously difficult to address in marine systems that lack geographic barriers to gene flow^[^ ^27^ ^]^.

In this study, we present chromosomal‐level genome assemblies for 10 pufferfish species, resolving long‐standing phylogenetic uncertainties and helping to more comprehensively understand the genetic differentiation associated with lineage specific phenotypes. Besides providing insight into evolutionary forces, the analyses of positive selection and introgression collectively identify candidate genes associated with lineage‐specific diversification, including inflation, coloration, and machinery, which contribute to genome stability. Our findings not only illuminate the genomic architecture of marine adaptive radiation but may offer insights for future genome engineering applications in vertebrates.

## 2 Results

### 2.1 Genome Assembly, Annotation, and TAD Organization

**Figure 1:**
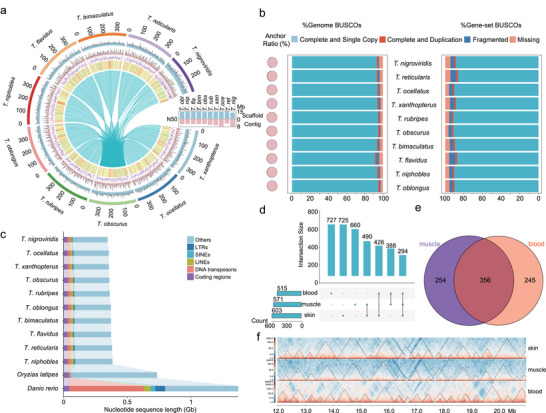
Characteristics and chromatin organization of pufferfish genomes. a) The synteny of each genome assembly against the reference *T. obs* (the inner bands), gene density, GC content, retrotransposon density, DNA transposon density, and the total length of each genome assembly (the most outer layer). The contig N50 (purple) and scaffold N50 (light blue) of each genome assembly are shown in a bar plot. b) The BUSCO evaluation on genome assemblies and gene sets are shown in the two middle bar plots for each species, with a moon phase represented on left side to indicate the ratio of chromosome anchoring. c) The total length of each genome assembly, dissected into coding regions, DNA transposons, retrotransposons (LINE, SINE, and LTR), and remaining non‐coding elements (Others). d) The number of shared topology association domains (TADs) from blood, muscle, and skin in *T. obs*. e) The overlapping TADs (shared domains) between muscle and blood samples in *T. bim*. f) An example of overtly conserved TADs with variation among skin, muscle, and blood in *T. obs*.

The whole genome assemblies of nine well‐characterized pufferfish species in the *Takifugu* genus and one green‐spotted pufferfish (*Tetraodon nigroviridis*) are reported here. A total of 1.4 million PacBio and 295.80 million Nanopore long‐reads were generated, resulting in an approximate coverage ranging from 43× to 141× for the sequenced species (Table S1, Supporting Information). To correct the error‐prone contigs assembled through long‐reads, high‐quality next‐generation sequencing short reads, on average 39.30 Gb per species, were subjected to the pilon pipeline to polish the genome (Table S1, Supporting Information). As a result, draft genome assemblies were obtained with a minimum scaffold N50 and contig N50 of 14.22 Mb and 4.71 Mb, respectively (Table S2, Supporting Information). With the polished genomes, 69.84 to 144.92 Gb (≈199 to 414×) Hi‐C data were collected to assist in the grouping and chaining of unanchored scaffolds into chromosomes (Figure S1, Supporting Information). This strategy generated chromosomal‐level genome assemblies with a size ranging from 346.48 to 385.03 Mb (**Figure** **1**a–c); average 98.07% of all bases were placed on chromosomes (Figure 1b; Table S2, Supporting Information). For *T. rubripes*, our assembly improved upon the existing reference (GCA_901000725.3) in contiguity (contig N50: 6.27 Mb versus 3.14 Mb), completeness (BUSCO intactness: 96.13% versus 95.22%), and chromosomal anchoring (99.59% versus 96.37%) (Figure 1b). Furthermore, to characterize genome organization among *Takifugu*, we identified topological associating domains (TADs) using Hi‐C data from 14 tissues across nine species. These TADs partitioned 79.54%–89.50% of each genome, with an average size of 510 kb and 622 TADs per species (Table S3, Supporting Information). Notably, only 54.50% of TAD boundaries were conserved across homologous chromosomes (Figure 1d–f; Table S4, Supporting Information), highlighting the divergence of genome organization within the genus *Takifugu*, despite its conserved genome size.

Gene annotation integrated 177.6 Gb of RNA‐seq data (25.37 Gb/species) with homology‐based predictions, yielding 19,846 protein‐coding genes per genome, on average. RNA‐seq support validated 93.91% of annotations, while BUSCO analysis confirmed 91.17% completeness (Figure 1b; Table S1, Supporting Information). Transposable element (TE) content was conservatively estimated at 15.48%–22.59% across species, with *T. nigroviridis* showing the highest TE density (Figure 1c; Table S5, Supporting Information). Specifically, DNA transposons, LINEs, SINEs, LTRs, and unknown elements constitute on average 8.07%, 8.85%, 0.36%, 5.92%, and 0.42% of the genome, respectively (Table S5, Supporting Information). Despite recent TE activity in *T. flavidus*, with 7.71% of TEs showing active within the last 5 million years (Figures S2, S3, Supporting Information), the total TE content remained stable—a pattern consistent with mechanisms that suppress TE accumulation in vertebrates.^[^ ^28^ ^]^ Comparative genomic analyses encompassing both cross‐species and intra‐species frameworks (newly assembled versus existing reference genomes) demonstrated conserved syntenic architectures with congruent TE and gene content patterns (Figure 1a,c; Figure S4, Supporting Information). These results validate the accuracy and utility of the assemblies for downstream analysis.

### 2.2 Functional Composition of the Most Compact Vertebrate Genomes

The reasons behind the diverse sizes of vertebrate genomes remain largely unknown. Using chromosome‐level genome assemblies of 10 pufferfish species, we conducted a comparative analysis of genome compactness, focusing on coding and non‐coding elements.

**Figure 2:**
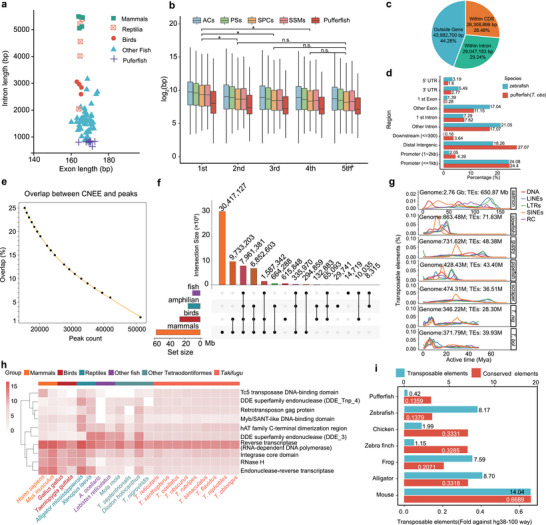
Maintaining the most compact vertebrate genome. a) The length statistics of exon and intron sequences extracted from 74 vertebrate genome assemblies. b) Box plot of intron sizes of each position from 5′ ends in teleost fish from a. Comparison between different position was conducted by Wilcoxin‐test in package ggpubr (v0.4.0; * denotes *p* <0.05). ACs: Anguilliformes, Acipenseriformes, Cypriniformes, Clupeiformes; PSs: Perciformes, Salmoniformes; SPCs: Scorpaeniformes, Synbranchiformes, Pleuronectiformes, Carangiformes, Cichliformes; SSMs: Mugiliformes, Gobiiformes, Osmeriformes, Syngnathiformes, Siluriformes.  c) Pie plot showing the distribution of highly conserved elements (HCEs). d) Bar plot showing the distribution of open chromatin distribution in different regions of the pufferfish (*T. obs*) and zebrafish genome. e) The overlap between CNEEs and open chromatin in *T. obs*. f) Conserved elements of different species identified by conserved elements in humans (100‐way, http://hgdownload.cse.ucsc.edu/goldenPath/hg38/phastCons100way/) as hit. g) Age of active transposable elements among various teleost fish. h) Heatmap of copy number of domains related to transposable element function. i) Volume of conserved elements and transposable elements of different species against conserved elements of humans (100‐way).

Gene structure evolution was assessed using 13,000 1:1 orthologs across 74 vertebrate genomes. Pufferfish exhibited the shortest introns among vertebrates (**Figure** **2**a), with middle introns consistently shorter than those at the 5′ ends of genes in fish (Wilcoxon test, *p* < 0.05, Figure 2b; Table S6, Supporting Information). Moreover, even excluding the impact of TE insertion, the lengths of the remaining intronic sequences between pufferfish and large vertebrate genomes showed only minor fluctuations (Figure S5, Supporting Information). This positional bias in intron length could be associated with transcript diversity, depending on alternative transcription start and termination sites^[^ ^29^, ^30^, ^31^ ^]^.

Non‐coding elements were analyzed through phylogenomic comparisons. The highly conserved elements (HCEs) in *Takifugu* exhibited a broad range in size, with 95% ranging from 40 to 1000 bp (Figure S6, Supporting Information). More than 55% of HCEs were located within gene regions, with 26.48% and 29.24% from coding and intronic sequences, respectively (Figure 2c). Cross‐species comparisons highlight differences in HCE genomic coverage. For example, HCEs in *Takifugu* spanned 25.21% of their genomes (92.62 Mb on average), compared to only 0.80% (10.95 Mb) in zebrafish and 4.38% (29.68 Mb) in medaka. These findings suggest that HCEs are required for genome functioning and hence cannot be lost despite the otherwise strong reduction in genome size.

In addition, conserved non‐exonic elements (CNEEs) occupied ≈70 Mb of sequence (Table S7, Supporting Information). ATAC‐seq profiling of *T. obscurus* liver, brain, and kidney revealed that more than half of open chromatin regions overlapped with CNEEs (Figure 2d,e; Figure S7, Supporting Information), implicating the importance of these elements to conserved regulatory functions. Cross‐species comparisons, however, revealed divergent regulatory landscapes. Zebrafish CNEEs showed higher enrichment in exonic/intronic regions compared to *Takifugu*, while the CNEEs in *Takifugu* are more likely located in the position of downstream, distal intergenic regions and promoter regions (Figure 2d). These observations are indicative of lineage‐specific regulatory rewiring mechanisms and selection.

To examine whether the regulatory rewiring and the compact genome were due to the loss of conserved elements in pufferfish, we compared their presence/absence across phyla. As expected, only a small proportion of the 100‐way conserved elements (phylum‐wide) were retained in pufferfish (Figure 2f). Interestingly, although most elements are specific to mammals, many lineages displayed independent increases or losses of conserved elements, highlighting massive lineage‐specific losses during vertebrate evolution.

### 2.3 Mechanisms Preserving Genome Compactness in *Takifugu*

To better understand which factors constrain genome expansion, we analysed TE insertion dynamics across 15 teleost lineages since their host genome split from their last most recent common ancestors (LMCAs) (Figure 2g; Figure S3, Supporting Information). In Atlantic salmon, common clownfish, and guppy, TE insertions occurred in multiple rounds, resulting in a non‐linear accumulation of TE sequences. Distinct classes of TEs, such as LTRs and LINEs in Atlantic salmon, SINEs in common clownfish and guppy, were selectively active before splitting from their LMCA. In contrast, SINEs in gulf pipefish, black scraper and *T. nigroviridis* were recently active after speciation. The expansion of distinct transcriptases, such as endonuclease‐reverse transcriptase in humans and DDE superfamily endonuclease (DDE_3) in frog, correlate with TE activity (Figure 2h). Notably, RNA retrotransposons in amniotes underwent massive expansion, whereas most DNA transposon expansion was subdued. The enriched transposition domains of active TEs further affected genomic composition (Figure 2g,h), suggesting that TEs may participate in re‐shaping regulatory sequences across vertebrates. The correlation between TE and genome size indicates that the net increase of TEs over time is likely determined by the propagation of transposon domains (Figure 2h; Figures S3, S8, Supporting Information). Therefore, TE insertion in compact genomes appears to be restrained in part because of fewer transposition enzyme domains.

While genome size is highly variable, it remains rather constant in several lineages of fish. Given TE accumulation cannot simply explain genome size expansion in some species, such as salmonids, cyprinids, and sturgeons, we examined conserved elements that independently evolved or became preserved in distinct lineages, particularly among teleost fish.^[^ ^32^ ^]^ Using 100‐way conserved elements as a baseline, we measured the ratio of conserved elements preserved, as well as the addition of newly evolved sequences in each lineage. *Takifugu* species lost about 85% of these elements (biased toward mammalian‐centric sequences), while birds retained ≈33%, confirming the lineage‐specific increase of conserved elements (Figure 2i). On the contrary, lineage‐specific sequence gain via TE insertion was 7–14‐fold higher in zebrafish, reptiles, and mammals than in *Takifugu*, in which the specific elements comprise a minor genomic fraction (Figure 2i). Lineage‐specific conserved elements were less commonly gained or newly evolved in certain fish species (Figure 2i).

In summary, this analysis revealed that the scarcity of transposon‐related domains in pufferfish genomes potentially contributes to their evolutionary stability and compact genome size (Figure 2h,i). These results collectively indicate that genome size evolution is a non‐linear process with sporadic increase and loss, entangled with waves of TE insertion.

### 2.4 Phylogenetic Relationships and Lineage‐Specific Introgression

**Figure 3:**
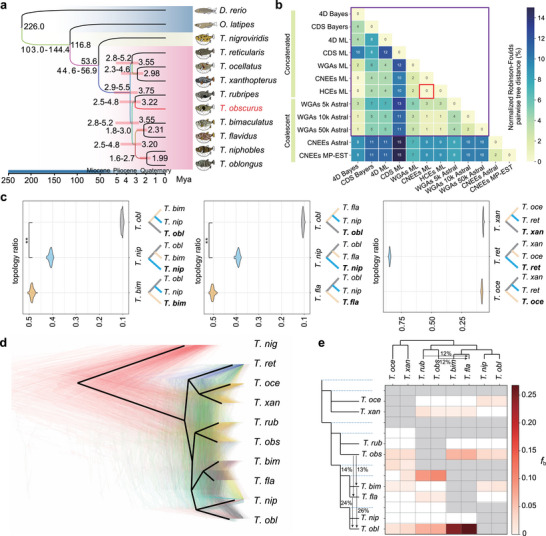
Phylogenetic relationships in the genus *Takifugu*. a) Time‐calibrated Astral phylogenetic tree of pufferfish, zebrafish and medaka based on a 5 kb sliding window of whole genome sequences. b) Summary of topology similarities, given as normalized Robinson–Foulds distances, between all phylogenies constructed in this study (phylogenies are shown in Figure S9 and Table S8; see also Table S8, Supporting Information for a summary of approaches and datasets). c) Evidence to distinguish between ILS and hybridization among three triplets (QuIBL output): *T. bim, T. nip, and T. obl*; *T. fla, T. nip, and T. obl*; and *T. ret, T. oce, and T. xan*. For each triplet, every species was set as an outgroup. The violin plots show the ratio of each triplet summarized from 100 QuIBL runs. Comparison between different topologies was conducted by Wilcoxin‐test in package ggpubr (v0.4.0; ** denotes *p* < 0.01). d) Cladogram with all nodes resolved in most species' trees (heavy black lines), where poorly resolved *Takifugu* clades collapsed as a polytomy. The 1000 colored trees were sampled from 10 kb non‐overlapping windows and constructed with maximum likelihood. e) The *f*‐branch (*f*_b_) statistic identifies gene flow from the branch of the tree on the *y*‐axis to the species or population on the *x*‐axis. The number next to the line with arrows shows the ratio of gene flow from one species to the other.

To resolve the phylogeny of *Takifugu* pufferfish, we integrated two complementary approaches: (1) concatenated whole genome/ortholog alignments and (2) multispecies coalescent, using *T. obs* as a reference for its high‐quality genome assembly. Both strategies converged on a consistent topology, with minor discrepancies limited to the placement of *T. obl* and *T. nip*; *T.oce* and *T. ret*. (**Figure** **3**a,b; Figure S9 and Table S8, Supporting Information). Crucially, 9 of 12 datasets supported nearly identical branching patterns, corroborated by independent analyses of four‐fold degenerate sites (4D Bayes), conserved non‐exonic elements (CNEEs ML), and highly conserved elements (HCEs ML) (Figure 3b; Figure S9, Supporting Information). Despite sporadic discordance in datasets involving 1–2 species, all trees consistently grouped species into two major clades. Except for a wider spreading of *T. obl*, some species in the two clades are distributed in the same region. Specifically, the first clade contains *T. rub, T. obs, T. bim, T. fla, T. nip*, and *T. obl*, the other clade that includes *T. ret, T. oce* and *T. xan* (Figures S9, S10, Supporting Information).

While introgression and incomplete lineage sorting (ILS) are known sources of phylogenetic conflict,^[^ ^33^, ^34^ ^]^ our analyses revealed that introgression disproportionately impacted three species: *T. obl, T. bim*, and *T. fla*. In the *T. obl‐T. bim‐T. fla* triplet, introgression affected 35.71% of loci—significantly more than ILS (4.06%; χ^2^ test, *p* < 0.05). In contrast, ILS dominated in the formation of *T. ret* (Figure 3c; Table S9, Supporting Information), suggesting complex evolutionary histories beyond simple bifurcation for species that diverged in the last 5 Million years. We reconstructed reticulate phylogenies using PhyloNet^[^ ^35^ ^]^ to examine whether these loci could result in non‐bifurcating relationships, identifying six hybridization events while recovering a consensus topology congruent with bifurcating trees (Figure 3d; Figure S11, Supporting Information). This consensus was further supported by CNEEs, HCEs and 4D‐based pairwise distances (Figure 3b), underscoring the robustness of our phylogenetic framework.

Additionally, 24.95 million high‐quality single‐nucleotide polymorphisms (SNPs) obtained from re‐sequencing data for 230 individuals across nine *Takifugu* species were used to elucidate evolutionary forces among *Takifugu* ^[^ ^36^ ^]^ (Tables S10–S12, Supporting Information). Using the population polymorphisms, ABBA‐BABA tests and *f*‐branch (*f*_b_) statistics confirmed widespread introgression, particularly in *T. obl, T. bim*, and *T. fla*, in agreement with the uncertainty in the reconstruction of their phylogenetic relationships (Figure 3e; Figure S12 and Table S13, Supporting Information). Independent analysis using HyDe (*p* < 0.05, Table S14, Supporting Information) reinforced these findings.

To this end, the phylogenetic relationship was comprehensively resolved with higher confidence using whole genome sequences. The relationship indicates *Takifugu* lineage underwent reticulate evolution and explosive speciation within the last 5 million years.^[^ ^5^ ^]^

### 2.5 Evolutionary Drivers of Population Divergence in *Takifugu*

**Figure 4:**
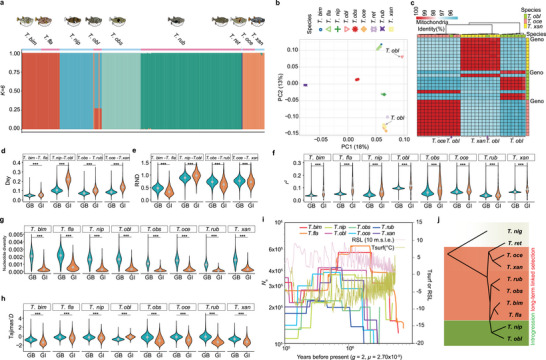
Complex evolutionary history of *Takifugu* speciation. a) Structure of *Takifugu* populations recovered from re‐sequencing data through Admixture analysis. Analysis with *K* = 6 gives the most robust clustering result. b) The PCA of 230 pufferfish individuals representing nine *Takifugu* species. c) Mitochondrial genome similarity heatmap among samples shows the complex compositions within the *T. obl* population. d–h) The Dxy, RND, linkage disequilibrium (*r^2^*), nucleotide diversity (Pi), and Tajima's *D* comparison between genomic islands (GI) and genomic background (GB) of close sister species. Wilcoxon test was performed; *p*‐values lower than 0.05, 0.01, and 0.001 are denoted by *, **, and ***, respectively. i) The estimated historical effective population sizes (*N*_e_) of *Takifugu* using SMC++. A generation time (*g*) of two years and mutation rate (*m*) of 2.7 × 10^−9^ substitutions per site per generation are used in the model. The relative sea level (RSL) reflected by 10 m sea level equivalent (m.s.l.e.) and atmospheric surface air temperature (Tsurf) are visualized in pink and green, respectively. j) The summarized mechanisms of evolution in pufferfish.

Population polymorphisms further allowed us to explore evolutionary mechanisms and population history of *Takifugu*. Population genomic analyses revealed six genetically distinct lineages within *Takifugu*, rather than the expected nine (**Figure** **4**a). Sister species exhibited near‐identical genetic backgrounds (except *T. nip* and *T. obl*; *T. rub* and *T. obs*), likely reflecting recent divergence insufficient to generate substantial genome‐wide differentiation. *T. obl* showed mixed population structure (Figure 4a,b), suggesting unresolved heterogeneity or potential homoploid hybridization between *T. nip* and *T. bim /T. fla* due to sympatric distribution (Figure S10, Supporting Information). Indeed, two phenotypically identified *T. obl* individuals, with discordant mitochondrial haplotypes, were excluded as putative hybrids (Figure 4c). They were excluded from subsequent analyses, since the remaining population primarily exhibited the difference of genetic background.

The population data were further employed to identify potential genomic loci contributing to speciation. SNPs of multiple populations were used to estimate fixation index (*F*_ST_), Tajima's *D*, nucleotide diversity *π*, and *D*‐statistics. Candidate genomic loci (genomic island of divergence, GI) were identified in four sister clades, resulting in 23.4 Mb from *T. bim* and *T. fla*, 16.1 Mb from *T. nip* and *T. obl*, 12.0 Mb from *T. rub* and *T. obs*, and 17.6 Mb from *T. oce* and *T. xan* sequences. In all four sister clades, GIs exhibited significantly higher genetic divergence (Dxy) than genomic backgrounds (GBs; Wilcoxon test, *p* < 0.05; Figure 4d). Further, We used the relative node depth (RND) to infer the potential source of divergence after considering the confounding effects of mutation rate. The RND analyses revealed distinct evolutionary trajectories: significantly lower RND of GI in three clades (*T. bim–T. fla; T. obs–T. rub;* *T. oce–T. xan*) implied long‐term linked selection, while higher RND of GI in *T. nip–T. obl* suggested introgression (Figure 4e). The higher nucleotide diversity observed in *T. obl*, which has a widespread distribution, suggests that it may have evolved more robust physiological traits to adapt to diverse habitats^[^ ^36^ ^]^ (Figures S10, S13, Supporting Information).

To uncover the potential driving force of evolutionary divergence between *T. nip* and *T. obl*, we tested gene flow and ancestral polymorphism differential sorting in both species using *D*‐statistics. We estimated *f*_4_ and *f*_b_ statistics to test for frequent introgression between *T. obl* and other species (Figure 3e; Figure S12, Supporting Information). The introgression ratio *f*_dM_ within GI showed that introgression was more pronounced in *T. obl* than in other species (Figure S13, Supporting Information). Long‐term linkage selection within GI substantially shaped diversification, as evidenced by a higher linkage disequilibrium index, *r* ^2^, compared to the background in all *Takifugu* species (*p* < 0.001), which is a sign of selection (Figure 4f).The decline in diversity within GI and adjacent sites suggests a strong genomic hitchhiking effect (Figure 4g), which is indicated by the Tajima's *D* pattern (Figure 4h). A significant hitchhiking effect introduced by stronger positive selection was confirmed by Fay and Wu's H test (Figure S14, Supporting Information). This evidence collectively highlights the prominent role that introgression played in the GI of *T. obl*, while strong selection occurred in sister species. Among these sister species, the lower *F*_ST_ between *T. bim* and *T. fla* suggests that these two species are in an early phase of speciation, with such a stage often accompanied by shift in population dynamics (Figure S15, Supporting Information). Our simulation of the effective population size of *Takifugu* revealed a rapid increase in *N*_e_, followed by decline during the last 5 million years (Figure 4i). This increase coincided with the timing of the Pliocene Messinian salinity crisis that altered the salinity of water within the *Takifugu* habitat (Figure 4i). The gene flow introduced by *T. obl* potentially has enhanced the fitness, reflecting by a recent increasing trend of *N*_e_.

In summary, the driving force of divergence in *T. xan* and *T. oce*, *T. obs* and *T. rub*, and *T. bim* and *T. fla* was subjected to long‐term linked selection. In contrast, *T. nip* and *T. obl* experienced introgression, which coincides with introgression‐dominated diversification in *T. obl* (Figure 4j).

### 2.6 Protein‐Coding Genes Underlying Lineage‐Specific Adaptations in *Takifugu*

*Takifugu* species inflate instantly in response to external threats, allowing them to escape vulnerable situations or to defend against predators—hence their name: pufferfish. This ability is thought to enhance their survival. Introducing acute mechanical stress through inflation can dramatically impact the physiological characteristics of pufferfish via activation of the molecular mechanisms involved in stress responses. To identify genes under positive selection linked to these traits, we analyzed orthologous genes using comparative evolutionary models (Experimental Section).

Using a two‐ratio branch model with all *Takifugu* species in the foreground, we identified hundreds of positively selected genes including *capn3*, *itga5*, *itga4*, etc., significantly enriched in integrin‐mediated signaling transduction as an example (Hypergeometric Test, *p* < 0.05, Figure S16, Supporting Information). Then the pufferfish as foreground was also used to detect positive selection genes, which are associated with cytoskeleton‐dependent intracellular transport (GO:0030705), cellular responses to DNA damage stimulus (GO:0006974), and microtubule‐based transport (GO:0099111), suggesting a tailored capability to cope with stress‐induced DNA damage (Figure S17, Supporting Information). A notable candidate is DNA polymerase theta (*polq*), which is critical for genome stability via microhomology‐mediated end joining and is a potential regulator of retrotransposon activity. Positive selection in *polq* suggests a dual role: repairing mechanical stress‐induced DNA damage and controlling mobile genetic elements, possibly influencing genome size.

Further investigation using a branch‐site model focusing on *Takifugu* species, as foreground, identified seven genes (*stom*, *trpv1*, *atp8b1*, *rhpn2*, *arhgap11a*, *pik3r1*, and *casq1a*) with lineage‐specific mutations that likely contribute to their unique adaptations. While four genes were found in the RHOA GTPase cycle (R‐DRE‐8980692), others were found to be involved in ion channel transport (R‐DRE‐983712) (Figure S18, Supporting Information). Our findings indicate that the mutation L177T in the prohibitin homolog of STOM is involved in regulating osmotic homeostasis, mechanosensation, and cell signaling^[^ ^37^ ^]^ (Figure S19, Supporting Information). The G605S change in the Pfam domain ion_trans of TRPV1 likely affects ion transport selectivity^[^ ^38^ ^]^ (Figure S19, Supporting Information). As the TRPV1 receptor is an essential sensor involved in desensitization to initiate anesthesia, the lineage‐specific mutation could be coupled with inflation's regulation of pain sensing. Moreover, the mutation Q460T of ATP8B1 is likely to affect microvillus formation in polarized epithelial cells,^[^ ^39^ ^]^ which could impact the morphology of cells during signal transduction (Figure S19, Supporting Information).

To validate the evolutionary specificity of these positive selection signals, we expanded our analyses to include more fish outgroups (*Cynoglossus semilaevis, Oreochromis niloticus*, and *Dicentrarchus labrax*), confirming positive selection at specific sites in *capn3* and *itga5* (Figure S20, Supporting Information). Functional modeling revealed that the asparagine‐to‐glycine substitution in CAPN3 likely modulates its catalytic activity, while the valine‐to‐tyrosine change in ITGA5 alters receptor binding properties^[^ ^40^ ^]^ (Figure S20, Supporting Information).

**Figure 5:**
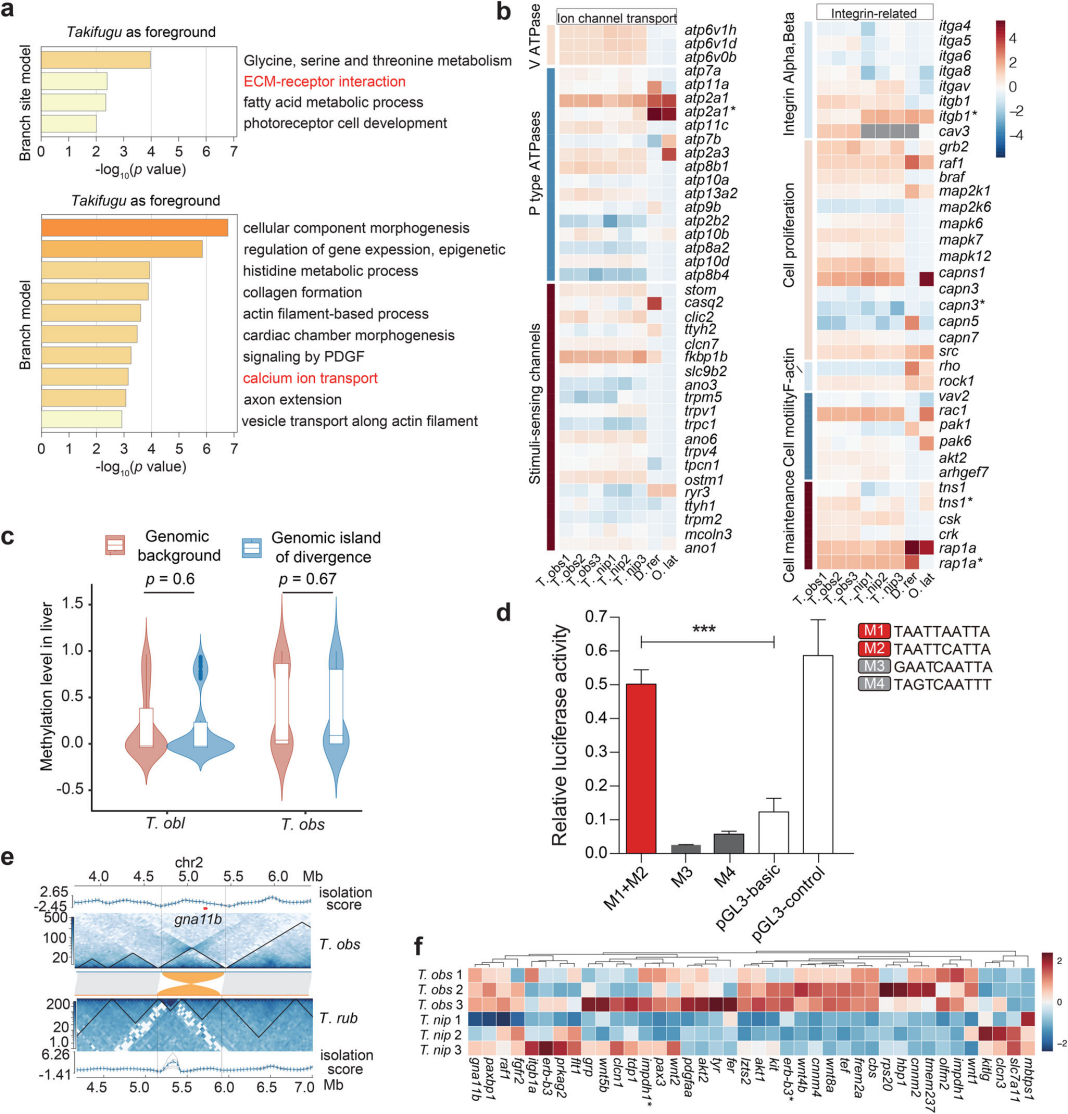
Genes associated with inflation and lineage‐specific coloration in *Takifugu*. a) Enrichment of positively selected genes in 10 pufferfish, using *T. nig* as an outgroup for the *Takifugu* lineage. b) Gene expression of ion channel transport and integrin‐related genes in fugu (with three replicates) and non‐inflatable fish species. The *z*‐score is individually calculated for each species. Genes marked with an asterisk (*) denote those present in multiple copies. c) Methylation level comparison between the genomic island of divergence and background of liver in *T. obs* and *T. obl*. d) A luciferase reporter assay was used to confirm the activity of pufferfish*‐*specific motifs in HEK293T cells. Four motifs, M1‐M4, within the candidate enhancer region upstream of *mitf* were cloned and transfected with or without *pax3* overexpression plasmid. Considering the physical proximity, three plasmids, carrying M1+M2, M3, and M4, were prepared individually. The vectors *pGL3‐Basic* and *pGL‐Control* represent the negative and positive control, respectively. One‐way ANOVA followed by Tukey's multiple comparison test was performed. Asterisks denote statistical significance (***, *p* < 0.001). e) An inversion‐introduced TAD variation that could have altered gene expression of essential gene *gna11b*, which is involved in melanin synthesis. The triangles at the top and bottom show the TAD of skin in *T. obs* and *T. rub*; the center part of the figure shows the synteny relation, while the grey and orange bands denote syntenic and inversion regions, respectively. f) The differentially expressed genes involved in coloration and pigmentation in the skin samples with two‐fold change in *T. nip* and *T. obs*. Genes marked with an asterisk (*) denote those present in multiple copies.

Genes under positive selection in *Takifugu* were enriched in the mechanosensation, including calcium ion transport and ECM‐receptor interactions which are linked to mechanical force sensing in human lung (**Figure** **5**a). This enrichment extended to ECM‐associated genes (*vtnb*, *lamb1a*), supporting systemic adaptations to repeated mechanical stress during inflation (Table S15, Supporting Information). Transcriptomic comparison of the expression profiles of genes related to ion channel transport and integrin in inflatable *Takifugu* compared to the non‐inflatable fish revealed an overall up‐regulation. An exception was a small number of ATPase genes that appear to be non‐symmetrically changed. These observations add support to the role of the rewired function of ion transporters, as pufferfish intake water or air into the stomach to inflate (Figure 5b).

By integrating open chromatin profiles with selection signals, we identified inflation‐associated regulatory elements located in the promoters of *elmo1*, *hapln2*, and *mapk13* and within the exon of *uhrf1bp1*. Interestingly, pufferfish‐specific mutations in MAPK13 (F98M, Y109F, V128I, M131T, A188G), a kinase regulating extracellular signal transduction, were linked to enhanced cellular stress responses^[^ ^41^ ^]^ (Figure S21, Supporting Information).

In addition to enrichment in mechanical force signaling and response, genes involved in fatty acid metabolism were also positively selected. It is likely that the *Takifugu* lineage adopted a distinct energy deposition/consumption strategy to enhance survival. CpG methylation profiling in liver revealed conserved methylation patterns within genomic islands of divergence (Figure 5c; Figure S22, Supporting Information), suggesting epigenetic regulation of lineage‐specific traits. Expression analyses further highlighted GI‐associated metabolic genes as hubs of regulatory innovation (Figure S22, Supporting Information). Together, these results delineate the molecular architecture of inflation, from force‐sensing pathways to metabolic adaptations.

### 2.7 Molecular Signatures of Speciation in Skin Patterning and Coloration

Skin coloration can be a key driver of reproductive isolation and speciation^[^ ^42^, ^43^, ^44^ ^]^ and exhibits striking diversity across *Takifugu* species. We identified positive selection in the melanogenesis‐related genes *pax3* and *tyr* within genomic islands (GIs) of *T*. *obl*, with *pax3* predicted to regulate *mitf*, a master melanocyte transcription factor (Figure S23, Supporting Information). Functional assays confirmed two *Takifugu*‐specific PAX3 binding motifs upstream of *mitf* that enhance transcriptional activity (Figure 5d), implicating them in melanin patterning. Species specific selection in *bco2* (*T. nip*) and *erb*‐*b3* (*T. xan*), which are linked to pigmentation diversity in vertebrates,^[^ ^45^, ^46^, ^47^, ^48^ ^]^ further underscores evolved coloration mechanisms (Figures S23, S24, Supporting Information).

We also identified inversions of more than 100 kb between *T. obs* and *T. rub* that could have rewired gene regulatory circuits due to chromatin reorganization. These inversions disrupted regulatory landscapes near six skin color‐associated genes (*gna11b*, *rps20*, *itgb1a*, *mbtps1*, *frem2a*, and *pdgfaa*). The experimentally validated inversion implies that such structural variation could have restrained the *gna11b* interaction with neighboring genes (Figure 5e; Figure S25, Supporting Information). Indeed, expression profiles of the skin transcriptome confirmed that pigmentation‐associated genes in *T. obs* are differentially expressed among sister species (Figure 5f). Quantitative PCR results further confirmed that *gna11b* was significantly upregulated in *T. obs*, thereby increasing the melanocyte expansion in *T. obs* versus *T. rub*, leading to higher melanin coverage (Figure 5f; Figure S25, Supporting Information). These findings reveal dual mechanisms— cis‐regulatory evolution and structural variation—that underlie rapid phenotypic diversification in *Takifugu* and highlight structural changes, such as inversion driving adaptive radiation,^[^ ^49^ ^]^ that support genomic plasticity.

## 3 Discussion

The compact genome architecture of *Takifugu* species provides a unique lens for studying vertebrate genome evolution. By integrating comparative genomics and population genetics, we identified molecular signatures of genetic differentiation, including lineage‐specific selection, structural variation, and regulatory rewiring, that disentangle the drivers of adaptive divergence. Our findings reveal how genomic plasticity enables rapid phenotypic innovation in compact genomes, offering broader insights into evolutionary mechanisms.

Despite decades of research, the mechanisms behind genome miniaturization remain enigmatic. We found that positive selection on POLQ, a DNA repair enzyme critical for TE regulation,^[^ ^50^, ^51^ ^]^ potentially underpins *Takifugu*’s genomic stability. By suppressing TE proliferation while enabling repair‐mediated integration of regulatory elements, POLQ balances the maintenance of sequence constraints and innovation of novel elements. This duality may explain the paradox of stable genome size, despite dynamic TE activity. Notably, recurrent loss of conserved elements and lineage‐specific TE expansions across fish genomes suggest that miniaturization is not a singular event, but a cyclical process shaped by evolutionary trade‐offs. A systematic and comprehensive comparison of small genomes and large genomes is expected to unravel additional evolutionary signatures.

The whole‐genome nuclear DNA sequences allowed us to overcome limitations of previous mitochondrial‐based studies.^[^ ^5^, ^52^ ^]^ Topological discrepancies reflect the mosaic histories of genomic loci, influenced by natural hybridization^[^ ^53^ ^]^ and ILS. Speciation events align with the Pliocene Messinian salinity crisis,^[^ ^54^, ^55^ ^]^ which likely affected adaptations to brackish habitats and *N*_e_ fluctuations. Such radiative diversification mirrors patterns in marsupials,^[^ ^56^ ^]^ birds,^[^ ^57^ ^]^ and fish,^[^ ^58^ ^]^ underscoring the universality^[^ ^27^ ^]^ of gene flow and ILS in marine vertebrates. Based on analyses of population data using this resolved phylogenetic relationship, we further uncovered two diversification mechanisms within GIs^[^ ^59^ ^]^: long‐term linked selection and introgression. These processes, also observed in cichlid fishes^[^ ^20^ ^]^ and butterflies,^[^ ^60^ ^]^ highlight the adaptive potential of compact genomes. While our study excludes some *Takifugu* species, the observed *N*_e_ dynamics and hybrid lineages provide a foundation for future work. Functional validation of lost conserved elements and engineering of adaptive loci could resolve mechanisms by which compact genomes balance stability with evolutionary potential.

Similar to the radiation of cichlid fish in isolated freshwater habitats, *Takifugu* have also undergone sympatric evolution.^[^ ^59^, ^61^ ^]^ However, *Takifugu* speciated in marine environments without obvious geographic barriers to gene flow, apparently spreading adaptive alleles across populations and balancing gene flow with local adaptation. With regard to the genetic basis of phenotypic diversification, we identified bona fide motif inclusion and structural variation that are responsible for diversified pigmentation in *Takifugu*. Our observations and experiments confirmed that these processes could rewire gene regulatory circuits. Motif inclusion and TAD rearrangement led to the confinement of cells responsible for skin coloration and patterning in *T. obscurus* and *T. rubripes*. As coloration is closely associated with mating, such variation from diverged individuals could potentially result in reproductive isolation and the emergence of new species. Identifying common mechanisms among species that have adapted to distinct environments may provide deeper insights into the features of forces driving speciation.

In summary, our multi‐omics approach helped deepen the understanding of mechanisms of genome evolution and speciation in *Takifugu*. By linking compact genomes to adaptive radiation through selection, structural variation, and regulatory innovation, we redefine miniaturization as a dynamic equilibrium of constraint and creativity. This framework invites exploration across vertebrates, from functional dissection to synthetic genome design.

## 4 Experimental Section

### Genome Analysis of Pufferfish

*Species sampling*: Nine *Takifugu* species and one green‐spotted puffer were initially selected to dissect phylogenetic relationships (Table S1, Supporting Information). DNA of all studied species were collected for genome sequencing, although genome assemblies of five species, including *T. rubripes*, *T. nigroviridis*, *T. flavidus*, *T. bimaculatus*, and *T. obscurus*, were published by other groups at the time of analysis. Various tissues, including muscle, skin, liver, gill, kidney, and eye, from seven species (*T. oblongus*, *T. niphobles*, *T. flavidus*, *T. obscurus*, *T. xanthopterus*, *T. ocellatus* and *T. nigroviridis*) were obtained for total RNA extraction. VAHTS mRNA‐seq v2 Library Prep Kit (Vazyme NR611‐02) was used following the manufacturer's instructions to prepare the library. DNA from intestine and liver of *T. bimaculatus*, *T. niphobles*, *T. oblongus*, *T. obscurus*, *T. ocellatus*, *T. rubripes*, and *T. xanthopterus* were extracted to perform whole genome Bisulfite‐sequencing (BS‐seq). All studied species were sacrificed under corresponding ethical permits and animal welfare guidelines.

### Sequencing

High molecular weight DNAs were subjected to standard genomic DNA sequencing library preparation for next‐generation sequencing (Illumina) and Nanopore sequencing. Following the manufacturer's specifications, Hi‐C libraries were prepared using the Dynabeads MyOne Streptavidin T1 (Invitrogen) kit and were sequenced on the DNBSEQ. RNA‐seq libraries of all fresh frozen tissues were prepared by capturing poly‐adenylated RNAs, which were then subjected to sequencing on DNBSEQ. The paired‐end libraries for BS‐seq were sequenced using Illumina HiSeq 2000 and 4000, according to the manufacturer's instructions. In addition, HMW gDNA of *T. obs* were prepared in a sequencing library for PacBio RSII to obtain ultra‐long reads to assist in the genome assembly process.

### Genome Assembly

*Construct the draft genome*. To construct high‐quality chromosomal‐level genome assemblies, a stepwise strategy was adapted to correct, assemble, and polish the genome assemblies. First, Canu (v1.7) was used to self‐correct the raw Nanopore reads, except for *T. obs* (Table S1, Supporting Information). For *T.obs*, the nanopore data (13.4G) and PacBio data (10.8G) were merged before feeding into Canu for self‐correction. Next, the corrected reads from the previous step were used to construct genome assembly by WTDBG (v1.28) with parameter‐set: –tidy‐reads 5000 ‐fo output.fasta ‐k 0 ‐p 21 ‐S 4. Finally, the newly assembled genome was further subjected to Pilon (v1.22, default parameter‐set) to polish the assemblies with more than 30× short reads for three rounds per species (Table S1, Supporting Information). The genome quality was evaluated through genome model BUSCO (v3.0.2) using the Odb9 Vertebrate set with zebrafish AUGUSTUS models and otherwise default parameters.

*Quality control of Hi‐C data*. First, reads were aligned with bowtie2 –very‐sensitive mode (v.2.2.5) to the draft genome assemblies. After filtering out duplicated and low‐quality reads by HiC‐Pro (v2.8.0), uniquely mapped reads were used to construct raw inter/intra‐chromosomal contact maps.

*Anchor Scaffold to Chromosomes*: Juicer (v.1.5) was used to generate interaction matrices at various resolutions. The 3D *de novo* assembly (3d‐dna, v. 170123) pipeline was then applied to organize scaffolds into chromosomes based on interaction frequency for each puffer genome. Scaffolds of each genome were organized and anchored to 22 pseudo‐chromosomes for *Takifugu* (21 for green spotted puffer), where 500 bp were introduced to fill the gap between contigs (Table S2, Supporting Information). The remaining scaffolds were kept as unplaced sequences.

### Mitochondrial Genome Construction and Comparison

To confirm the sampling of correct species, NOVOPlasty (v3.2) was used to assemble the full‐length mitochondrial genome of each resequencing sample (Table S10, Supporting Information). Then FastANI was applied to estimate the whole‐genome average nucleotide identity and generate the mitochondrial similarity matrix to compare the similarity of each sample and subsequent visualization.

### Repeat Annotation

Homology‐based and *de novo* prediction strategies were carried out to annotate the repetitive elements. In detail, RepeatMasker (v4.0.5) and RepeatProteinMasker (v4.0.5)^[^ ^62^ ^]^ were used to detect interspersed repeats and low complexity sequences against the Repbase database (21.01)^[^ ^63^ ^]^ at both nuclear and protein levels, respectively. Then, RepeatMasker was used to detect species‐specific repeat elements using a custom database generated by RepeatModeler (v1.0.8)^[^ ^64^ ^]^ and LTR‐FINDER (v1.0.6).^[^ ^65^ ^]^ Moreover, Tandem Repeat Finder (v4.0.7)^[^ ^66^ ^]^ was employed to predict tandem repeats. All predicted repeated annotations were integrated and merged into one repeat annotation.

### Gene Model Annotation

Repetitive sequences were masked prior to gene model annotation. Several approaches, including *de novo* gene prediction, RNA‐seq based annotation, and homology‐based annotation, were applied to predict and/or annotate genomes before merging all evidence into one combined non‐redundant annotation.

### Homolog‐Based Gene Annotation

Protein sequences of well‐annotated genes from nine species were downloaded as homologous query sequences from Ensembl (release 93); the species included *Homo sapiens, Danio rerio, Oreochromis niloticus, Gasterosteus_aculeatus, Gadus morhua, Gallus gallus, Oryzias latipes, Takifugu rubripes*, and *Tetraodon nigroviridis*. To identify matched regions, query protein sequences were aligned to all masked genomes with BLAT (Kent, 2002). GeneWise (v2.2.0)^[^ ^67^ ^]^ was used to continue searching for accurate alignments at the target regions and to predict homolog‐based gene models.

### *De Novo* Gene Prediction

AUGUSTUS (v3.1)^[^ ^68^ ^]^ was employed to scan the whole genome using a custom training set generated by selecting the longest 2000 high‐quality genes model from the transcriptome‐based methods.

### RNA‐Seq‐Based Annotation

The transcriptome reads of all tissues obtained from corresponding species (Table S1, Supporting Information) were mapped to the newly assembled genome with HISAT2.^[^ ^69^ ^]^ The mapped reads were subjected to StringTie^[^ ^69^ ^]^ to assemble gene transcripts. TransDecoder (v5.5.0, https://github.com/TransDecoder/TransDecoder) was then applied to predict the candidate complete ORFs and generate the gene models supported by expression profiles.

### Combine the Gene Model

To obtain a consensus gene set, EvidenceModeler software (EVM, v1.1.1) was used to integrate gene models generated through RNA‐seq based, homology‐based, and *de novo* prediction evidence with the following weight setting: ABINITIO_PREDICTION: PROTEIN: TRANSCRIPT = 1:2:10. Afterward, PASApipeline (v2.3.3) was applied to complete UTR annotation of gene models using RNA‐seq data. Genes with short‐length (<50 aa) and premature stop codons were removed from the consensus gene set (Table S2, Supporting Information).

Functional annotation of predicted protein‐coding gene models was performed by aligning the coding regions to sequences in public protein databases with BLAST (ncbi‐blast‐2.6.0+), including NCBI nonredundant protein (NR, 20 200 204), Kyoto Encyclopedia of Genes and Genomes (KEGG, 87.0), Eukaryotic Orthologous Groups of proteins (KOG), and SwissProt databases (202301). Gene Ontology annotation was also performed with EggNog (v5.0).

### ATAC‐Seq Peak Calling

Raw sequencing reads were subjected to quality control followed by a filtering process with SOAPnuke with parameter‐set ‘‐A 0.2 ‐M 2 ‐l 10 ‐q 0.1 ‐n 0.05 ‐Q 2′. Clean reads were mapped to the *T. obscurus* (reference) genome using Bowtie2 (v.2‐2.3.4.1) with parameter‐set very‐sensitive and the resulting alignments were sorted by SAMtools (v1.9) according to coordination. MACS2 was utilized for peak calling using the following pipeline to identify consistent peaks between biological replicates for a given tissue: a relaxed significance threshold (*p* ≤ 0.05) was utilized for each sample, which was followed by a strict cut‐off (*q* ≤0.05); peaks were called again using both libraries for each bio replication. The peaks from the relaxed run were then filtered to the same strict cut‐off (*q* ≤ 0.05) used in the pooled runs, and the pooled peaks were linked to the filtered peaks from each library. Peak overlap greater than 0 was then extracted to perform the downstream analysis, producing the final peak for each sample (Table S16, Supporting Information). Further overlapping analysis among various region is conducted by BEDTools.

### Topology Association Domain

*Reads Mapping*: Reads of pair‐end reads were aligned to the genome by Bowtie2 (v.2.3.4.1), with parameter‐set –local –reorder. The resulting alignment was converted into bam format by SAMtools (v1.9).

*Creation of a Hi‐C matrix*: The hicBuildMatrix function of HiCExplorer (v2.2.3)^[^ ^70^ ^]^ was then used to construct the matrix of read counts of more than 5 kb bin in the genome, considering the sites around the given restriction site.

*Merge Bins for the Hi‐C Matrix*: Next, the hicMergeMatrixBins (v2.2.3) was applied to merge the 5 kb bins matrix into 40 kb bins.

*Correction of Hi‐C matrix*: One histogram showing the sum of contact per bin (row sum) were produced with hicCorrectMatrix (v2.2.3) diagnostic_plot function. The contact distribution per bin was inspected to determine the reasonable threshold for removing bins with fewer reads. Last, the Hi‐C matrix was corrected with hicCorrectMatrix (v2.2.3) function with the appropriate threshold.

*TAD Calling*: The hicFindTADs was used to identify TAD domains and boundaries in the corrected Hi‐C matrix at a resolution of 40 kb (Table S3, Supporting Information).

*Visualisation of TAD*: The Hi‐C heatmap and putative TADs were visualised in Juicebox ^[^ ^60^ ^]^ and hicPlotTADs in HiCExplorer software.

### Comparative Genomic Analysis of Pufferfish

*Gene Family Analysis*: The protein sequences of 10 pufferfish and outgroup species, including medaka, zebrafish, mouse and human (ENSEMBL 93), were clustered with SonicParanoid.^[^ ^71^ ^]^ The reciprocal best hit (RBH) genes with 1:1 orthologous relationship among all 14 species were generated using blastp function in ncbi‐blast (v2.7.1+). The gene family expansion and contraction were then evaluated using CAFE v5.0^[^ ^72^ ^]^ with results from the SonicParanoid pipeline. A conditional *p*‐value was calculated for each gene family, and families with conditional *p*‐values smaller than 0.05 were considered to have undergone a significantly accelerated rate of expansion or contraction. To estimate the divergence time, 4d sites from 6049 ortholog pairs were extracted and fed into MCMCTree in PAML v4.9e package^[^ ^73^ ^]^ for the estimation of divergence time with a calibration based on fossil records from the TimeTree website.

### Comparison of Gene Structure Among Various Organisms

Using RBH methods, the gene sets of 74 species, including mammals, birds, reptiles, fish (Table S6, Supporting Information), were collected for the generation of a 1:1 orthologous dataset. The average exon and intron length was estimated from the annotation file of each genome. We also isolated the fish groups and divided their intron positions into five categories for further comparison. The length comparison of intron among different positions was conducted by Wilcoxin‐test in package ggpubr (v0.4.0; * denotes *p* < 0.05).

### Phylogeny Analysis of the *Takifugu* Genus

*CDS tree (Mrbayes, ML)*: The 4D sites from 10,084 one‐to‐one orthologous genes were extracted. In total, 26,406,729 sites were catenated and used to construct a Maximum Likelihood (ML) phylogenetic tree by RAxML using the default parameters. A total of 1,000 bootstrap replicates were performed to calculate the support for each node (Figure 2b; Table S8, Supporting Information).

*4D Tree (Mrbayes, ML)*: A total of 2,318,510 4d sites were extracted from 10,084 one‐to‐one orthologous genes and catenated. Next, RAxML was used to estimate an ML phylogenetic tree with default parameters. One thousand bootstrap replicates were performed to calculate the reliability of each topology (Table S8, Supporting Information).

*Whole Genome Alignment Tree (WGA tree)*: The multiple species alignment generated in the above step was used to construct the phylogeny analyses to resolve phylogenetic relationships among pufferfish. To generate high‐quality alignment (225,113,720 bp), poorly aligned sites were discarded based on the number of species (less than four species in the alignment). The filtered alignment was fed to RAxML with the GTRGAMMA model, and 100 rapid bootstraps were used to construct the WGA tree (Table S8, Supporting Information).

*Astral Tree (Whole Genome Alignments Tree)*: In the Astral tree‐building process, the WGA was first divided into 5, 10, and 50 kb windows by Bedtools. The phylogenetic tree of each window was estimated with the same method described in the WGA tree. Next, 10 000 windows of trees were randomly selected as the input for Astral to obtain the consensus topology (Table S8, Supporting Information).

*HCE Tree*: HCE generated by the previous step (79,635,025 bp) were refined by filtering out the poorly aligned sites, followed by feeding into RAxML to reconstruct the phylogenetic tree with a GTRGAMMA model and 100 rapid bootstraps (Table S8, Supporting Information).

*CNEE Tree*: The CNEEs tree was constructed using the same approach described above for the HCE tree (42,820,660 bp) (Table S8, Supporting Information).

*Network Species Tree*: To test the existence of a reticulated phylogenetic relationship, analyses were carried out for *two major clades in Takifugu*, the *T. obscurus* clade (*T. rubripes*, *T. niphobles*, *T. oblongus*, *T. bimaculatus*, and *T. flavidus*) and the *T. reticularis* clade (*T. nigroviridis*, *T. xanthopterus*, *T. ocellatus*, *T. reticularis*, *T. rubripes*. and *T. obscurus*). Analyses were performed using an approach similar to the one described in the butterfly study.^[^ ^60^ ^]^

### Positive Selection Analysis

Two datasets were employed to detect positively selected genes to capture *Takifugu*‐specific and pufferfish‐specific signals. The pufferfish dataset included 10 species of fish, specifically nine *Takifugu* and one *Tetraodon*. The other dataset consisted of 12 fish species, which were included in the pufferfish, medaka, and zebrafish datasets. A total of 6,049 one‐to‐one single‐copy genes were used to detect positive selection signals. For the pufferfish dataset, every *Takifugu* fish had a foreground branch, with the other nine pufferfish as the background branch. The branch‐site likelihood ratio test was performed using codeml's free ratio model in PAML. Genes with *p*‐values less than 0.05 were considered as candidates for positive selection. For the 12 fish dataset, we conducted the branch‐site likelihood ratio test with *Takifugu* as the foreground branch and the remaining three species as the background branch.

### Construction of Multiple Species Alignment

*Pairwise alignment*: The repetitive sequences, except tandem repeats in the chromosome‐level genome, were soft‐masked by Bedtools (v2.27.1). Pairwise alignments were built using LASTZ‐1.04.00, using *T.obs* as the target genome, The parameter for closely related species was selected to achieve better alignment among following *Takifugu*: *T. bim, T. nip, T. fla, T. oce, T. ret, T. xan, T. nip, T. rub*, which was the same set used between human and chimpanzee ( https://asia.ensembl.org/info/genome/compara/mlss.html?mlss = 1098). However, due to the distant relationship, a parameter set for distantly related species was applied to align *T.obs* and *T.nig* (https://asia.ensembl.org/info/genome/compara/mlss.html?mlss = 1160). The alignments were further processed to the best‐aligned blocks using UCSC tools for “chaining” and “netting”.

*Multiple Sequence Alignment*: MULTIZ (v11.2) was used to aggregate pairwise alignments into multiple genome alignments, with the *T. obs* genome as reference. The MULTIZ shell scripts were generated by ROAST software (v3) using the phylogenetic tree (based on the 4D tree) as a guide. Approximately 336.62 Mb syntenic sequences shared by all studied pufferfish genomes and the outgroup species, the *T. nig*, were obtained in final alignment (Table S7, Supporting Information). A similar procedure was applied for alignments among 12 species, and 358.16 Mb syntenic sequences were kept.

### Conservation Analyses

*HCE*: A neutral substitution model was built through PhyloFit (v1.4, “REV” substitution model) using 4D sites of *T. obs* protein‐coding sequences. Highly conserved elements in *T.obs* were predicted by PhastCons with parameter‐set –target‐coverage 0.3 –expected‐length 45 –rho 0.3, and the neutral substitution model was built by PhyloFit (Table S7, Supporting Information).

*CNEEs*: CNEE was obtained by excluding the CDS region from the HCE. The “subtract” function in Bedtools was applied to the HCE identified above to remove CDS to obtain the candidate CNEE. Next, candidate CNEE sequences of *T. obs* were extracted and used as a query to search against the genomes of zebrafish, medaka, and *Takifgu* by BLAST (ncbi‐blast‐2.6.0+). All hits with more than 50% coverage with target sequence and e‐value less than 1e‐5 were excluded. Finally, candidate CNEE regions shorter than 30 bps were discarded to generate the final CNEE set (Table S7, Supporting Information).

### Vertebrate Conserved Elements Lost in *Takifugu*

For inference of ancestral vertebrate HCEs losses in multiple organisms, especially for puffers, HCEs datasets for Homo sapiens (hg38) were downloaded from phastConsElements100way of UCSC, further it was excluded the conserved elements shorter than 30 bp, After filtering 1,171,885 conserved elements was left, accounting for 87.9 Mb, these vertebrate CNEs were then searched in the mammals (mouse), birds (chicken, zebra finch), reptiles (frog, alligator) and fish (puffers and zebrafish) using BLAST v2.7.1 (evalue = 1e‐6, word size = 6, max target seqs = 1, max hsps = 1).

### Estimation of Active Age of Transposable Elements Among Various Organisms

It was selected five fish (Atlantic salmon, common clown fish, guppy, and gulf pipefish) with mutation rates from Bergeron et. al,^[^ ^74^ ^]^ along with one Black Scraper (*T. sep*), and then re‐annotated their genomes to identify repetitive sequences. To do so, the same pipeline it had previously used in pufferfish species was employed. It was also used a custom Perl script parseRM.pl (https://github.com/4ureliek/Parsing‐RepeatMasker‐Outputs) to estimate the TE activities of each genome based on alignment outputs from RepeatMasker. The result of the analysis was packed into a bin per 0.5 MYA. In addition, we first retrieved the divergence times for all species involved from the TimeTree website (https://timetree.org/), the divergence times as a reference age to analyze the activity of TEs before and after the divergence events.

### Genome‐Wide Protein Domain Annotation

To depict the genetic basis for TE expansion, we annotated all Pfam‐A protein domains from the Pfam genome database. All these genomes were collected from open‐access databases, such as NCBI and Ensembl. It was translated each genome with the 6‐phase model using EMBOSS v6.5.7 and searched domains across the genomes using HMMER v3.1b2 with default parameters. Furthermore, the domains related to previously reported was analyzed ^[^ ^65^ ^]^ TEs expansion in pufferfish and outgroup species, including human, mouse, chicken, zebra finch, alligator, frog, common clownfish, guppy, ocean sunfish, greenfin horse‐faced filefish, freckled and porcupinefish. The corresponding domains in all these species were counted to reflect TE activity (Figure 3h).

### BS‐Seq Analysis and Methylation Level Calculation

Sequence reads were pre‐processed using Trim Galore to remove adapter contamination and low‐quality bases on both ends. The clean reads were aligned to the reference genome using Bismark. Multiple reads mapping to the same position were regarded as PCR duplicates, and only one was included. Bases with a quality score < 20 were filtered for subsequent analysis. The error rate of each library (sum of the nonconversion rate and T/C sequencing errors) was calculated as the total number of sequenced Cs divided by the total sequencing depth for sites corresponding to Cs in the Lambda genome. The error rate for each library was 0.5%. To distinguish true mCs from false positives. The methylation level of an individual CpG was determined by the number of reads containing a C at the site of interest divided by the total number of reads covering the site. The methylation level of a specific region was determined by the sum of methylation levels of individual CpGs divided by the total number of covered CpGs.

### Population Genetics Analysis of the *Takifugu* Genus

*Data Collection and Genotyping Calling*: Publicly available resequencing data of 173 samples representing eight pufferfish from multiple datasets, including PRJDB12458 (17 samples), PRJNA638440 (66 samples), PRJNA522329 (90 samples), and the newly generated data (57 samples) (Figure 4a), were collected for population analyses. The raw sequencing reads were trimmed by Fastp (v0.20.0) with the default parameter setting (Tables S10‐S12, Supporting Information). To accelerate reads mapping and SNP detection, Sentieon Genomics Tools (202010.02) was used for mapping, sorting, duplicate removal, re‐alignment, haplotype calling (for each sample), and joint calling (for populations), with *T.obs* as the reference genome. Next, a series of filtering steps were carried out to generate a high‐quality SNP dataset. We first applied GATK53 (v3.8.1) VariantFiltration to filter SNPs with a standard hard filter criterion of ‘QD < 2.0 || MQ < 40.0 || FS > 60.0 || ReadPosRankSum < ‐8.0 || MQRankSum < ‐12.5 || SOR > 3.0′, according to the GATK manual. Second, polymorphic sites with more than two alleles, likely produced by ambiguous alignment, were also removed. The filtering procedures resulted in 24945865 SNPs (Table S11, Supporting Information).

### Analysis of Population Structure and Parameter

To conduct population structure analysis, a prune SNP dataset (3,342,301 bi‐allelic SNPs) was generated using PLINK (v1.90b6.6) with parameters ‘–indep‐pairwise 50 10 0.2′ to avoid bias due to linkage disequilibrium. The individuals with a sequence depth of less than 10× were also excluded to avoid bias due to low sequencing depth. STUCTRUE (v2.3.4) was used to perform unsupervised ancestral component analysis, with the K value (number of assumed ancestral components) ranging from 1 to 8 with different seeds by the parameter of “‐D”. A principal component analysis (PCA) was performed in PLINK with default parameters, and the result was visualized in R. To estimate the population parameter, all sites with missing data and minimum quality score < 30 were discarded to obtain a missing‐free high‐quality SNP dataset, the output dataset contains 16,631,733 SNPs. Then a customized script ‘popgenWindows.py’ (https://github.com/simonhmartin/genomics_general) was employed to calculate *F*_ST_, *θ*π, and dxy values in 10 kb non‐overlapping sliding window across the genome (Tables S17 and S18, Supporting Information).

### Estimation of the Mutation Rate per Generation for Pufferfish

To estimate the mutation rate per generation for pufferfish, the gene sets of Atlantic herring and zebrafish were clustered, thereby obtaining the gene pair between these two species. These gene pairs were then bridged to pufferfish through the ortholog relationship between zebrafish and pufferfish. Bridging enabled us to gain the gene pair between Atlantic herring and pufferfish, resulting in 6,289 usable gene pairs. Next, the median value of Ks of 6,289 genes of Atlantic herring and pufferfish was determined by KaKs_Calculator (knowing that the mutation rate of Atlantic herring is 2.0 × 10^−9^ ^[^ ^28^ ^]^). The following formula was used to estimate the mutation rate: Ks (Atlantic herring)/Ks (puffers) = Mutation rate(Atlantic herring)/Mutation rate (puffers) (Table S19, Supporting Information).

### Gene‐Flow Analysis

*Takifugu Diverged Recently*: Here, two types of analyses were carried out to detect gene flow between species. ABBA‐BABA‐based analysis was conducted using Dsuite and Dfoil. Another strategy relying on the phylogenetic tree, QulBL, was applied to distinguish ILS (Incomplete Linkage Sorting) and gene flow to infer evolutionary history.

*Dsuite Analysis*: The high‐quality bi‐allelic SNPs (16,631,733 bp) dataset was used to detect gene flow in the *Takifugu* genus, with *T.ret* as the outgroup, using the “Dtrios” command in Dsuite with default parameters. The result was summarized in Table S13 (Supporting Information).

*QulBL Analysis*: The topology of 27,519 qualified loci was inferred from 279.5 Mb realigned WGAs as the candidate input set for QuIBL analysis.^[^ ^49^ ^]^ Five thousand individual trees were randomly selected from this set as input for one QulBL run, and 100 runs were conducted to make the result stable and reboot (Table S9, Supporting Information).

1) 5000 individual trees from this candidate set were randomly selected as an input for one QuIBL estimation, and this random selection procedure was repeated 100 times to generate 100 independent QuIBL outputs.

2) QuIBL then calculated the likelihood values (Bayesian Information Criterion test, BIC). The inner branch lengths in Subset 2 and Subset 3 were best described by a simple exponential distribution as expected under ILS (scenario 1) or a mixture of ILS and introgression (scenario 2). The difference in BIC values (Delta. BIC) was calculated as the BIC value of scenario two minus the BIC value of scenario 1. Since the BIC value is less than 0 when Delta.BIC is greater than 10, the scenario of ILS only with the lower BIC value is preferable. However, when Delta.BIC is less than ‐10, the scenario of a mixture of ILS and introgression with the lower BIC value is preferable. In other cases, the two scenarios are indistinguishable.

3) QuIBL also inferred the theoretical distributions of inner branches under ILS or introgression for Subset 2 and Subset 3. After plotting these two theoretical distributions, they could be compared visually with the observed distribution of inner branches. All 100 QuIBL outputs were summarized in Table S9 (Supporting Information).

### HyDe Analysis

To test the potential hybridization between species and search for molecular evidence, the inheritance parameter (γ) was estimated using HyDe. Specifically, 176,866,368 sites containing only one individual were employed to test whether one of the species in the triplet could be the hybrid offspring of the remaining two species (Table S14, Supporting Information).

### Plasmid Construction and Motif Activity Analysis In Vitro

Based on the *Takifugu rubripes* genomic sequencing data, the primer pair M1+M2‐luc‐F/R and M3‐luc‐F/R were designed for amplification and sequence validation. For these pairs, the XhoI and HindIII recognition sites and *pGL3‐basic* vector (Promega, Madison, WI, USA) homology arm sequence were added to the 5′ ends of each forward and reverse primer, respectively. M4‐luc‐F/R was designed for amplification and validation, with XhoI and HindIII recognition sites added to the 5′ ends of forward and reverse primer, respectively (Table S20, Supporting Information). The *pGL3‐basic* vector was cleaved with corresponding enzymes and ligated with purified PCR product of M1+M2 and M3 by ClonExpress Ultra One Step Cloning Kit (Vazyme, Nanjing, China); this produced the *pM1+M2‐pGL3‐basic* (*M1+M2*), *pM3‐pGL3‐basic* (*M3*) plasmids, respectively. The purified PCR product of M4 and *pGL3‐basic* vector were cleaved with corresponding enzymes and ligated with T4 DNA ligase (Yugong, Jiangsu, China) to obtain the *pM4‐pGL3‐basic* (M4) plasmid. The ORFs of *pax3* were amplified and cloned into the HindIII and EcoRI identification sites of *pcDNA3.1(+)* (Invitrogen, Carlsbad, CA, USA) to obtain *pcDNA3.1‐pax3* (*pax3*). The vector *pGL3‐basic* was set as negative control, while *pGL3‐control* was set as positive control. An EndoFree Plasmid Mini Kit (TIANGEN, Beijing, China) was used to prepare all plasmids.

Human embryonal kidney (HEK) 293T cells used in the study were purchased from the Shanghai Institute of Cell Biology. Cells were cultured at 37 °C with 5% CO_2_ in DMEM medium (Solarbio, Beijing, China) supplemented with 10% fetal bovine serum (FBS) (Gibco, Carlsbad, CA, USA), 800 IU mL^−1^ penicillin, and 800 µg mL^−1^ streptomycin (Solarbio, Beijing, China). For the dual‐luciferase analysis, 500 ng of *pM1+M2‐pGL3‐basic* (*M1+M2*), *pM3‐pGL3‐basic* (*M3*), or *pM4‐pGL3‐basic* (M4) were transfected or co‐transfected with 500 ng of *pcDNA3.1‐pax3* (*pax3*) into HEK293T cells. An amount of 40 ng *pRL‐TK* plasmid was also co‐transfected into each well. After co‐transfection using Lipofectamine 3000 Reagent (Invitrogen, Carlsbad, CA, USA) for 48 h, cells were collected and tested by the Dual‐Luciferase Reporter Assay System (Promega, Madison, WI, USA) and Infinite 200 PRO Multimode Microplate Reader (TECAN, Männedorf, Switzerland). Each experiment was performed in triplicate. Relative luciferase activities (the ratio of firefly luciferase to renilla luciferase) were shown as mean ± SEM (n = 3). One‐way ANOVA followed by Tukey's multiple comparison test was used for analysis.

## Conflict of Interest

The authors declare no conflict of interest.

## Author Contributions

Q.W. as a co‐first author, who contributed equally with K.L. C.S., J.T. and G.F. conceived and designed the project. J.Z. and K.L. analyzed data. J.Z., K.L. and M.S. interpreted results. J.Z, K.L. and C.S. wrote the manuscript. N.W prepared NGS sequencing libraries. M.T. assisted NGS sequencing. L. M., S. L., and S.H. assistance in bioinformatics. H. W., S. L., Y. L., L.B.D., W. L., and M.Z. collected and prepared samples. Q. W., Y. Z., and Q. L. performed the luciferase reporter assay and q‐PCR experiments. Y.Q. performed ATAC‐seq experiments. S.P. performed Hi‐C experiments. A. M., J. T., M. S. and C. S. reviewed and edited the original draft.

## Supporting information



Supporting Information

Supporting Information

## Data Availability

All sequencing data generated by this study have been deposited into the China National GeneBank DataBase (CNP0004688).
