# Supplementary material for: Patterns and Processes of Genomic Evolution Inferred From the Ten Smallest Vertebrate Genomes
Source: Adv Sci (Weinh). 2025 Sep 10;12(38):e17251. doi: 10.1002/advs.202417251 (PMC12520575; doi:10.1002/advs.202417251)
Supplement: Supplementary file 1 — Supporting Information [file ADVS-12-e17251-s001.docx]

Supporting information for

**Patterns and processes of genomic evolution inferred from the ten smallest vertebrate genomes**

Contents

[1. Genome assembly, TAD comparison and genome quality assessment 3](#_Toc205918587)

[2. Conserved elements analysis 4](#_Toc205918588)

[3. Phylogenetic relationship construction 5](#_Toc205918589)

[4. Mitochondria similarity analysis 6](#_Toc205918590)

[Supporting Information figures 8](#_Toc205918591)

[Supporting Information tables 33](#_Toc205918592)

# Genome assembly, TAD comparison and genome quality assessment

Firstly, we filtered raw sequencing data by discarding low-quality reads (defined as >10% bases with quality values less than 10 and >5% unidentified (N) bases), adaptor-contaminated reads and PCR duplicate reads. We trimmed a few bases at the start and end of reads according to the FastQC (v0.11.2) results. Secondly, we used WTDBG to perform the initial assembly with corrected long reads with parameters “--tidy-reads 5000 -fo output.fasta -k 0 -p 21 -S 4”. Thirdly, to obtain a chromosome-level genome, HIC-Pro(v2.8.0) (Servant et al., 2015) was used for quality control of Hi-C sequencing data with parameters [BOWTIE2_GLOBAL_OPTIONS = --verysensitive -L 30 --score-min L,-0.6,-0.2 --end-to-end –reorder;BOWTIE2_LOCAL_OPTIONS = --very-sensitive -L 20 --score-min L,-0.6,-0.2 --end-to-end –reorder; IGATION_SITE = GATC; MIN_FRAG_SIZE = 100; MAX_FRAG_SIZE = 100000; MIN_INSERT_SIZE = 50; MAX_INSERT_SIZE = 1500]. Finally, the software packages Juicer (Durand et al., 2016) and 3d-dna (v170123) (Dudchenko et al., 2017) were employed to generate contact matrices of chromatin and constructed chromosomes with parameter [-m haploid -s 0 -c 22/21 (22 for *Takifugu*, 21 for *T. nig*)] based on the karyotype information supplied previously. Notably, the percentage of unplaced scaffolds was only between 0.24% and 1.75% except for *T. ret* (4.48%) in *Takifugu* and 9.97% for green spotted pufferfish (**Figure 1b;** **Figure S1, Table S2**)

TAD boundaries were directly compared across distinct tissues within the same species. For cross-species TAD comparison, lift-over was used to converted coordinates to the reference *T. obs* genome, then compared with the same rule as within the same species.

To systematically evaluate the quality of our genome assemblies, we have conducted analyses on the following three aspects: completeness, accuracy, and contamination. (1) To assess genome completeness, we also conducted reads alignments using both NGS and TGS reads, besides the widely used BUSCO (v3.0.2) completeness score (an average score for studied species: 95.34%, **Figure 1b**). These concordant results—spanning gene-space completeness and read representation—robustly validate the high quality and completeness of our genome assembly. (2) To demonstrate the accuracy of the genome assemblies before and after error correction, we further performed analyses using Merqury (v.1.3) (Rhie et al., 2020). The average score increased from 21 (18.06-27.52, pre-correction) to 30 (28.01-33.02, post-correction). This indicated that base-level error rate decreased from 0.9% to 0.1% after correction. These findings collectively reflect that the error correction process has substantially enhanced the overall accuracy of genome assemblies. (3) We have indeed checked the potential contamination since this is a common procedure. However, the initial GC-depth analysis demonstrated a uniform GC content distribution, indicating well-controlled contamination (**Figure S1**). To further strengthen this conclusion, we also performed a contamination check using FastQ-Screen (Wingett & Andrews, 2018) and found no or extremely low contamination. These findings indicate the absence of significant contamination in the genome.

# Conserved elements analysis

We speculated that conserved elements had independently evolved or preserved in distinct lineages, particularly in teleost fish. By using 100-way conserved elements as a baseline, we measured the ratio of preserved conserved elements and the increase in each lineage, and found that the ratio of conserved elements in fish lineages was relatively stable and much lower compared to reptiles, birds and mammals. Considering that 100-way alignment were highly biased towards mammalian genomes, the relative fold change of genomic sequences introduced by conserved elements and transposons was employed to illustrate the size variation across lineages. For instance, fugu missed about 85% of conserved elements, which is mainly due to the bias towards mammalian genomes. In contrast, birds and reptiles contained one-third of the conserved elements, confirming the lineage-specific increase (**Figure 2i**). Meanwhile, the TE expansion showed variable dominating roles to reshape the genome size, with an 7 to 14-fold efficiency compared to conserved elements, particularly in zebrafish, reptiles and mammals. However, the fish specific elements only occupied a small proportion, indicating the lineage specific gain of conserved elements in distinct lineages and newly evolved elements in fish species. The drastically fewer transposition related domains could be one of the main factors that determined the compact genome size of pufferfish (**Figure 2g, h**). CNEEs were considered overlapping if they covered >=10% of the open chromatin peak length as examined by BEDTools (v2.26.0).

# Phylogenetic relationship construction

In the concatenated strategy, several datasets, including whole genome alignments (WGAs), highly conserved elements (HCEs), conserved non-exonic elements (CNEEs) and orthologous protein-coding orthologous genes (CDS), were employed to infer the topology of a phylogenetic tree using either Maximum Likelihood (ML) or Bayesian algorithms (**Table S8**). For the multiple-species coalescent strategy, WGAs and CNEEs datasets at multiple resolutions (window size: 5kb, 10kb and 50kb) were implemented to resolve the phylogenetic relationships by jointly using Astral and MP-EST. Results from these two strategies almost concordantly supported a resolved topology for the *Takifugu* species (**Figure 3a**), despite a minor discrepancy in the placement of *T. obl* and *T. ret* (**Figure S9**). Among the evidence derived from multiple datasets, the shared topology inferred by 4-fold degenerate site (4D) (Bayes), CNEE (ML) and HCE (ML) were chosen to represent the consensus topology of the species tree based on the observation that 9 out of 12 datasets provided by both strategies displayed nearly identical topologies (**Figure 3b**).

Finally, to resolve the observed minor discrepancy leading to the misplacement of *T. obl* and *T. ret*, triplet sub-tree analyses were carried out to infer the most probable topology in each branch individually (**Figure 3c; Figure S9 and Methods**). Briefly, the topology of genomic loci at 5 kb, 10 kb, and 50 kb resolution for each triplet was summarized and statistically tested to obtain the most strongly supported topologies. The subtree analysis and other evidence supported that CNEE represents the consensus topology as indicated by the minimum pairwise distance serving a dissimilarity index (**Figure 3b**).

# Mitochondria similarity analysis

A meticulous sequence comparison of mitochondria genomes of all investigated individuals was carried out to gain alternative evidence. As the sequences of the DNA barcoding gene Cytochrome C Oxidase subunit I (COI) of these species were non-informative due to their nearly identical sequences, whole mitochondria sequences were used to confirm species identity (**Figure 4c**). In line with the STRUCTURE, species *T. obl* also presented heterogeneous maternal genomic composition (**Figure 4b**) once excluded the two ambiguous *T. obl* individuals, reinforcing the existence of compound genomic compositions in *T. obl*. These results provided evidence of frequent gene flow (**Figure 3e**).

**Reference**

Durand, N.C., Shamim, M.S., Machol, I., et al. (2016). Juicer provides a one-click system for analyzing loop-resolution Hi-C experiments. *Cell Syst.* 3, 95–98.

Servant, N., Varoquaux, N., Lajoie, B.R. et al. (2015). HiC-Pro: an optimized and flexible pipeline for Hi-C data processing. *Genome Biol.,* 16, 259.

Dudchenko, O., Batra, S. S., Omer, A. D., et al. (2017). *De novo* assembly of the Aedes aegypti genome using Hi-C yields chromosome-length scaffolds. Science, 356(6333), 92–95.

Rhie, A., Walenz, B. P., Koren, S., et al. (2020). Merqury: reference-free quality, completeness, and phasing assessment for genome assemblies. *Genome Biol.*, 21(1), 245.

Wingett, S. W., & Andrews, S. (2018). FastQ Screen: A tool for multi-genome mapping and quality control. *F1000Res*., 7, 1338.

# Supporting Information figures


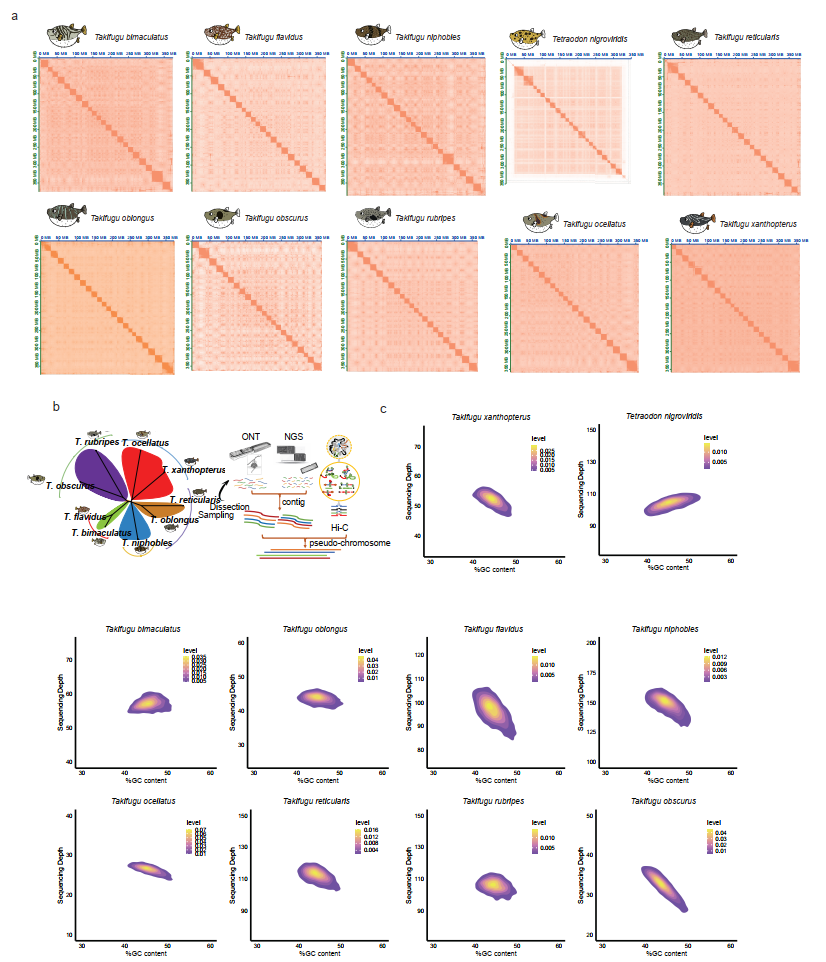


**Figure S1 | Genome assembly of 10 pufferfish genome.** **a,** Hi-C contact heatmap of the chromosomes, scaffolds of the pufferfish genome assembly. **b,** The flowchart of genome assembly step. ONT denotes Oxford Nanopore Technology, NGS denotes Next-generation sequencing, Hi-C denotes High-throughput/resolution chromosome conformation capture. **c,** GC-depth of pufferfish genome, the horizontal axis of the figure represents GC content, and the vertical axis represents sequencing depth. The large central plot is a scatter plot based on the GC content and coverage depth information of the contigs, where the color scale reflects the density of the plotted points.


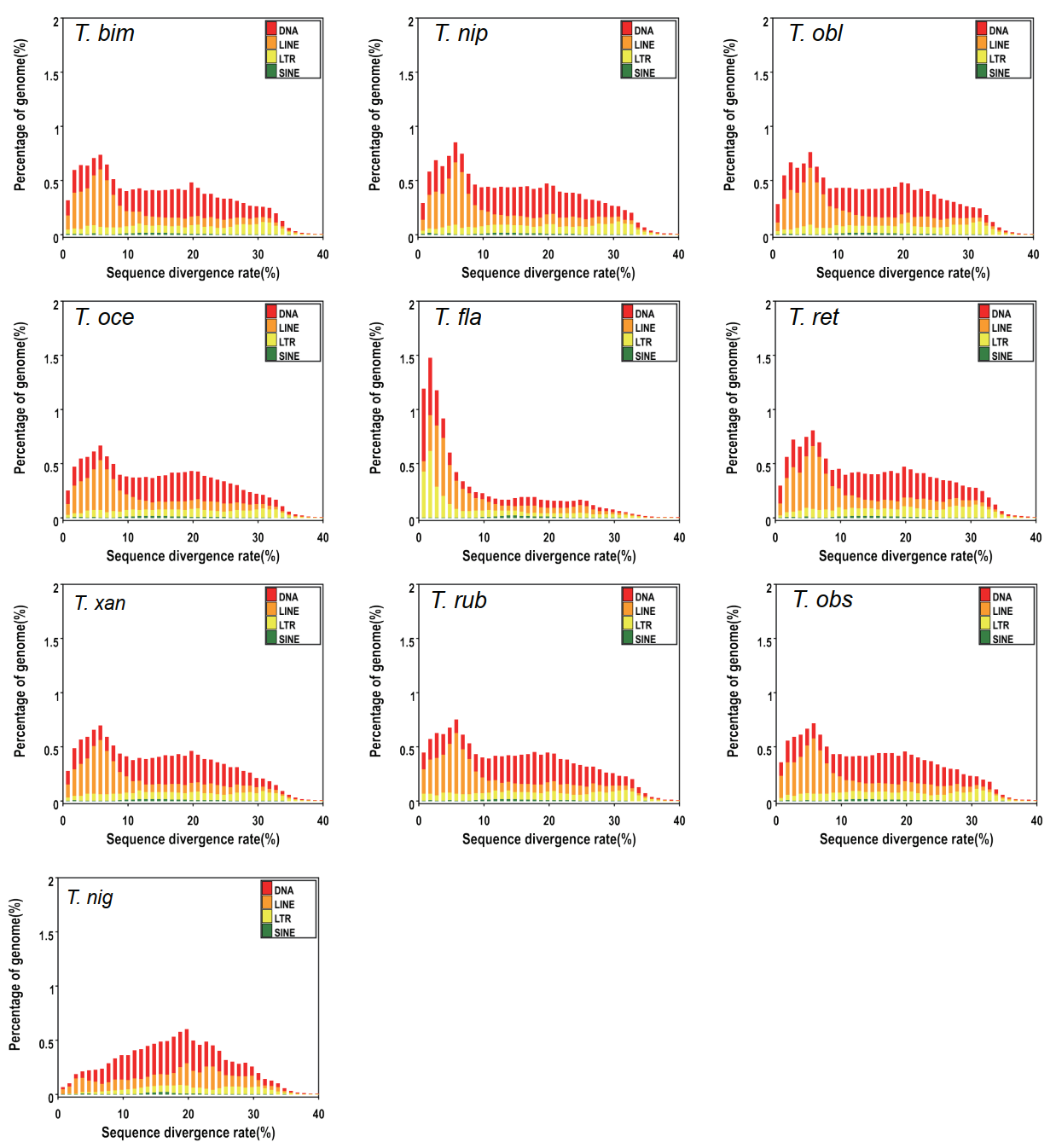


**Figure S2 | Distribution of sequence divergence rate of each type of TEs in the ten pufferfish genome.** The divergence rate was calculated between the identified TE elements in the genome and the consensus sequence in the TE library used Repbase (v202101).

**
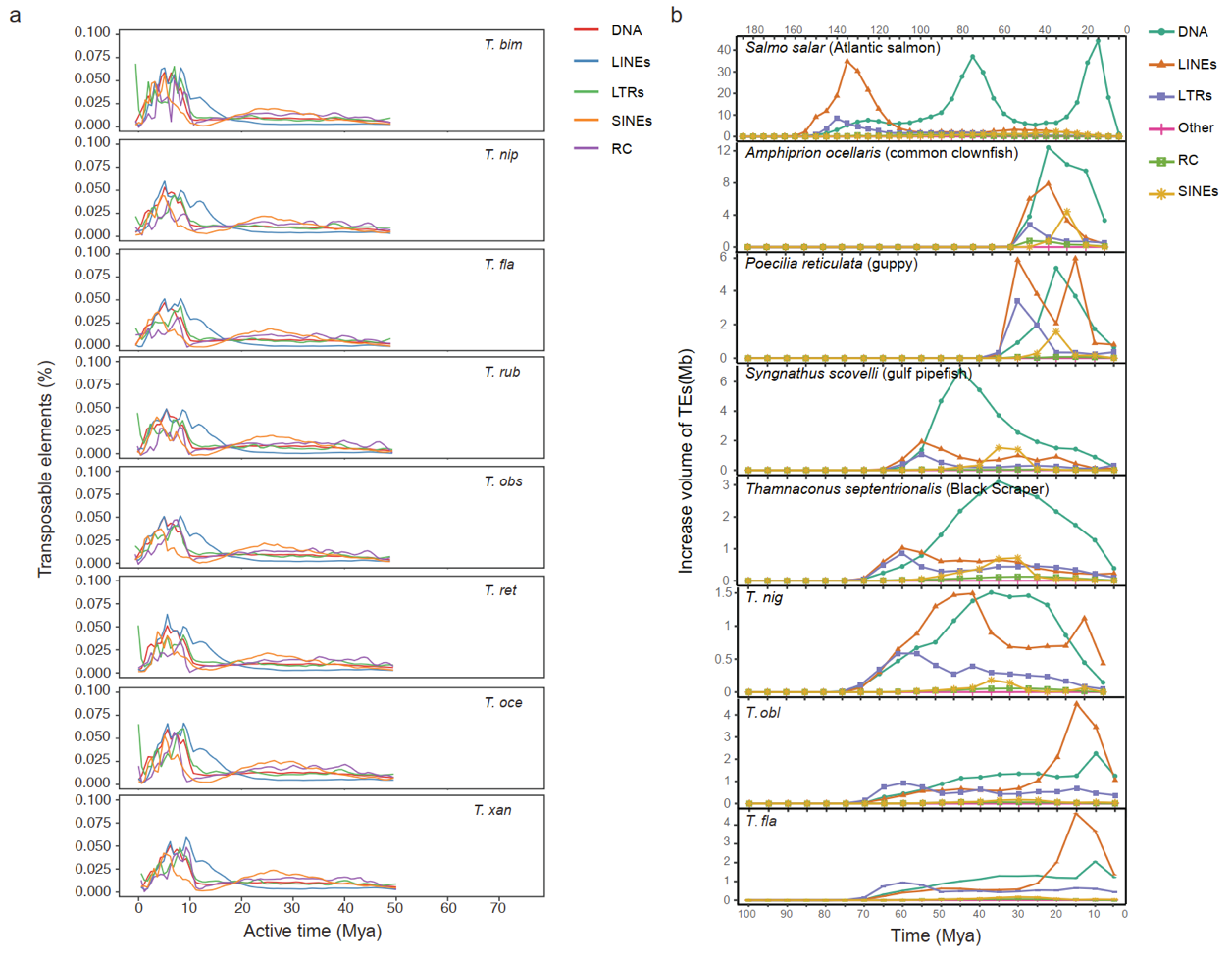
**

**Figure S3 | TE activity along the history. a.** Age of active transposable elements among various fish. **b.** Line plot of increasement of TEs along the evolutionary history among various fish genomes.


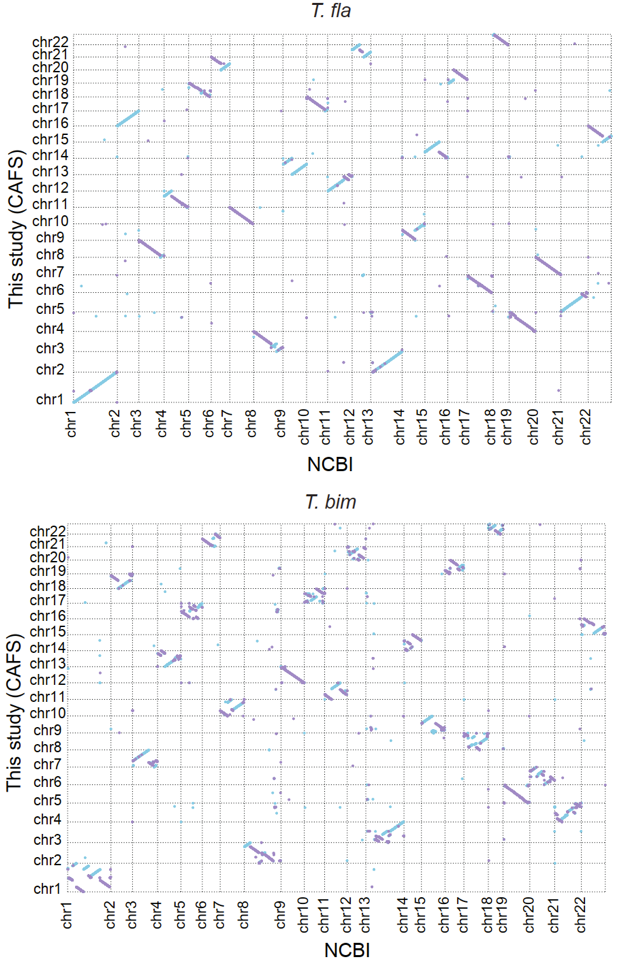


**Figure S4 | Dot plots of syntenic conservation between published *Takifugu* genome (x axis) and our version (y axis).**


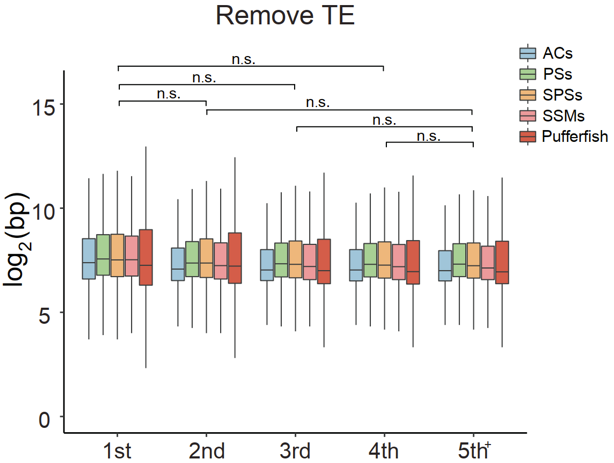


**Figure S5 | Box plot of intron sizes of each position from 5’ ends in teleost fish after removing TE.** Comparison between different position was conducted by Wilcoxin-test in package ggpubr **(**v0.4.0; Wilcoxon test, n.s. denotes *p* > 0.05).


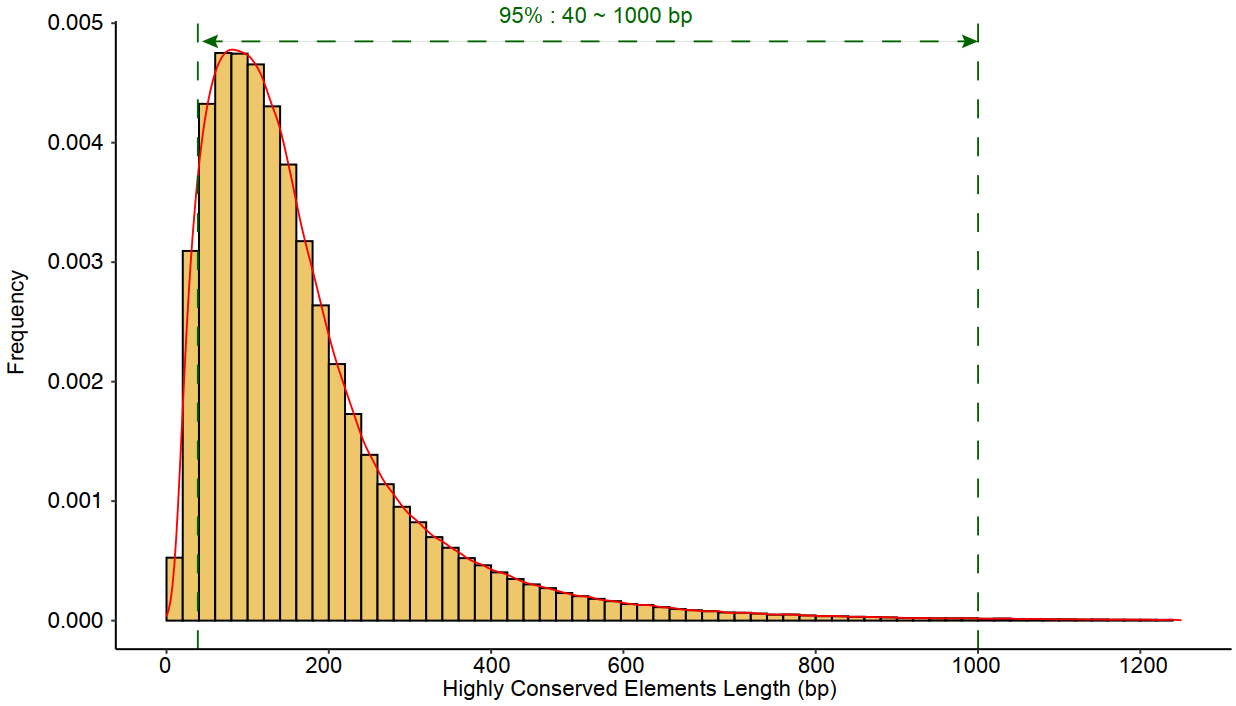


**Figure S6 | The length distribution of highly conserved elements (HCEs)**.


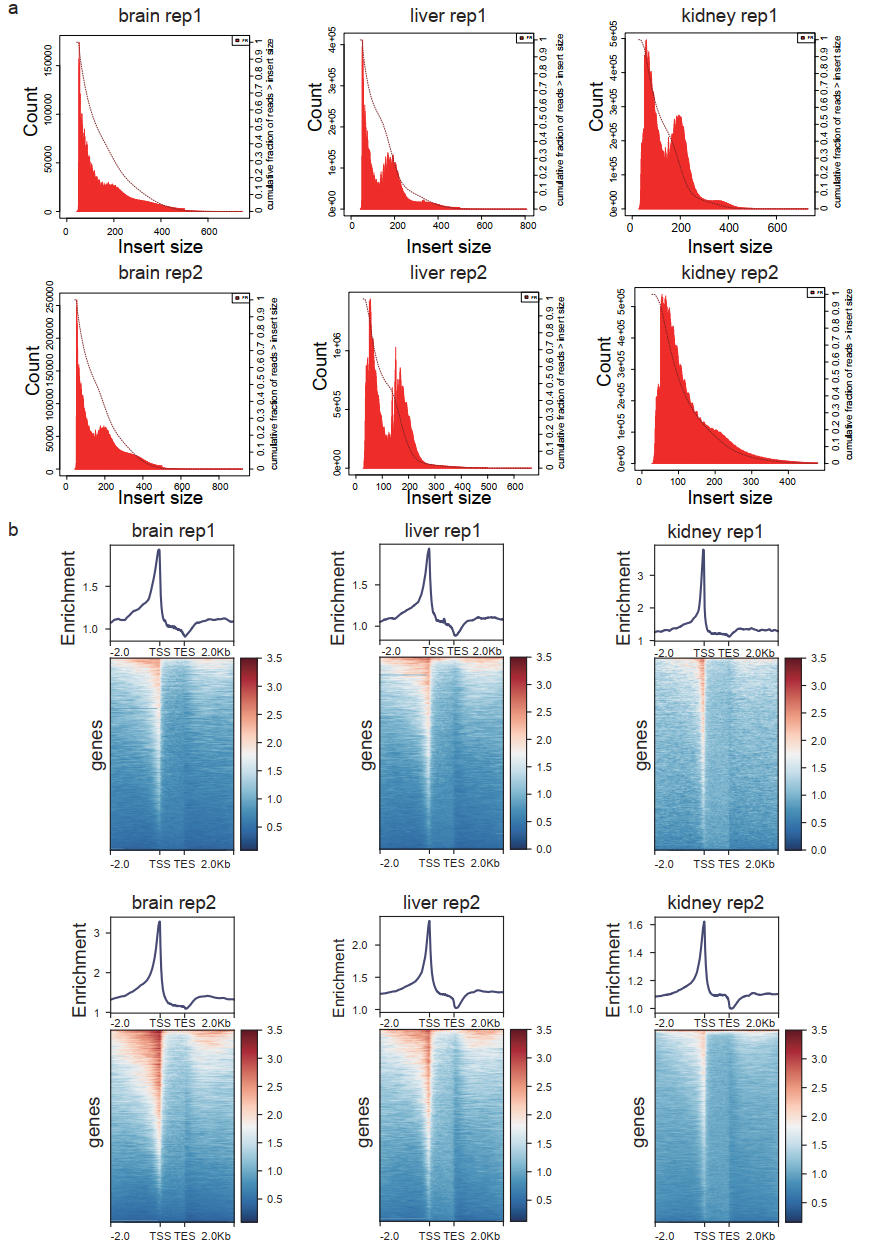


**Figure S7 | ATAC-seq quality control.** **a,** Fragment size distribution for each sample. **b,** ATAC-seq signal is enriched at transcription start sites (TSS) and transcription end sites (TES) of genes.

**
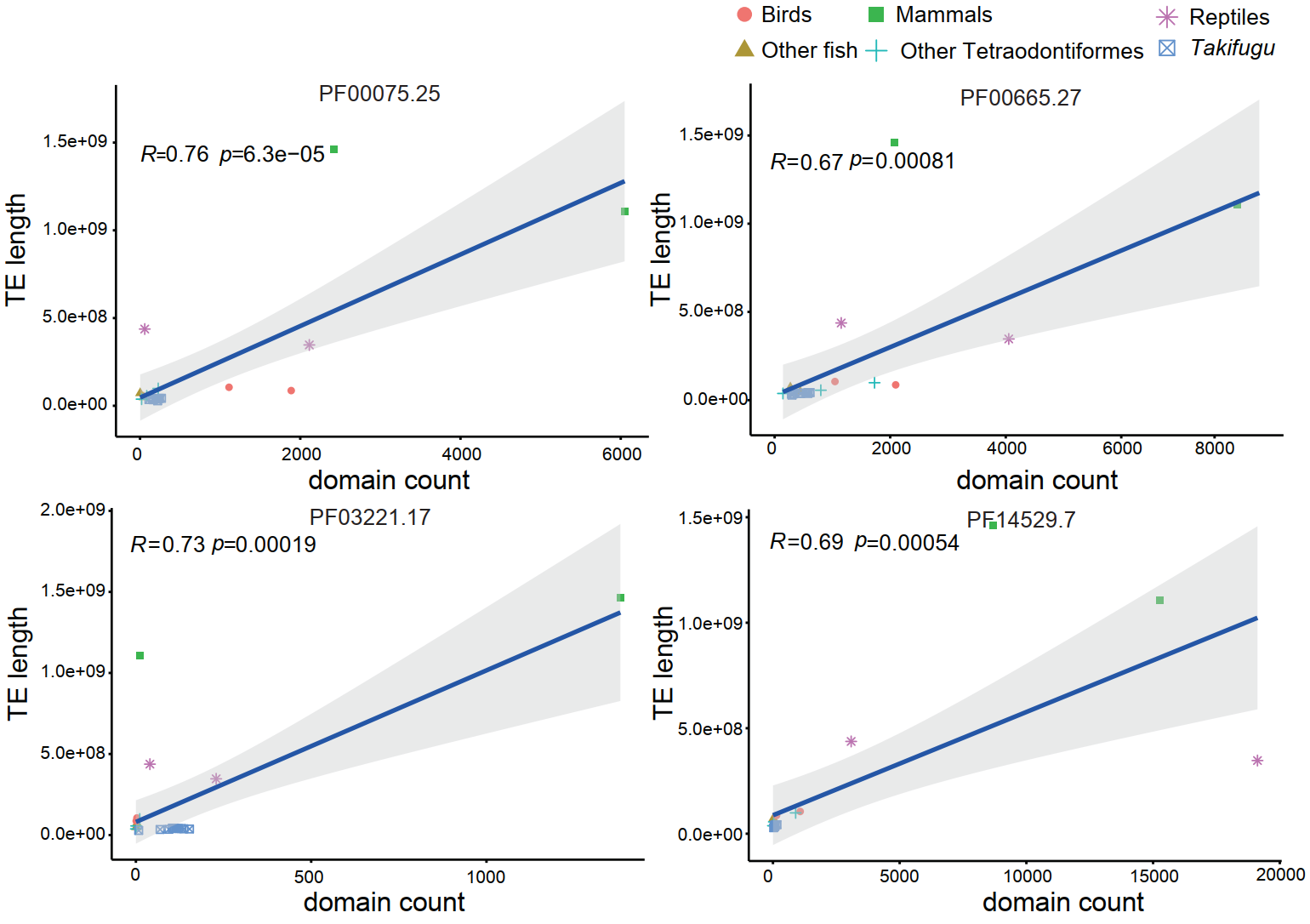
**

**Figure S8 | Pearson correlation between the count of TE-related domains (x axis) and TE length (y axis) among the teleost genomes.** Pearson correlation analysis was performed by cor.test function in R, and *p*-values lower than 0.05 was significantly correlated between TE-related domains and TE length.

**
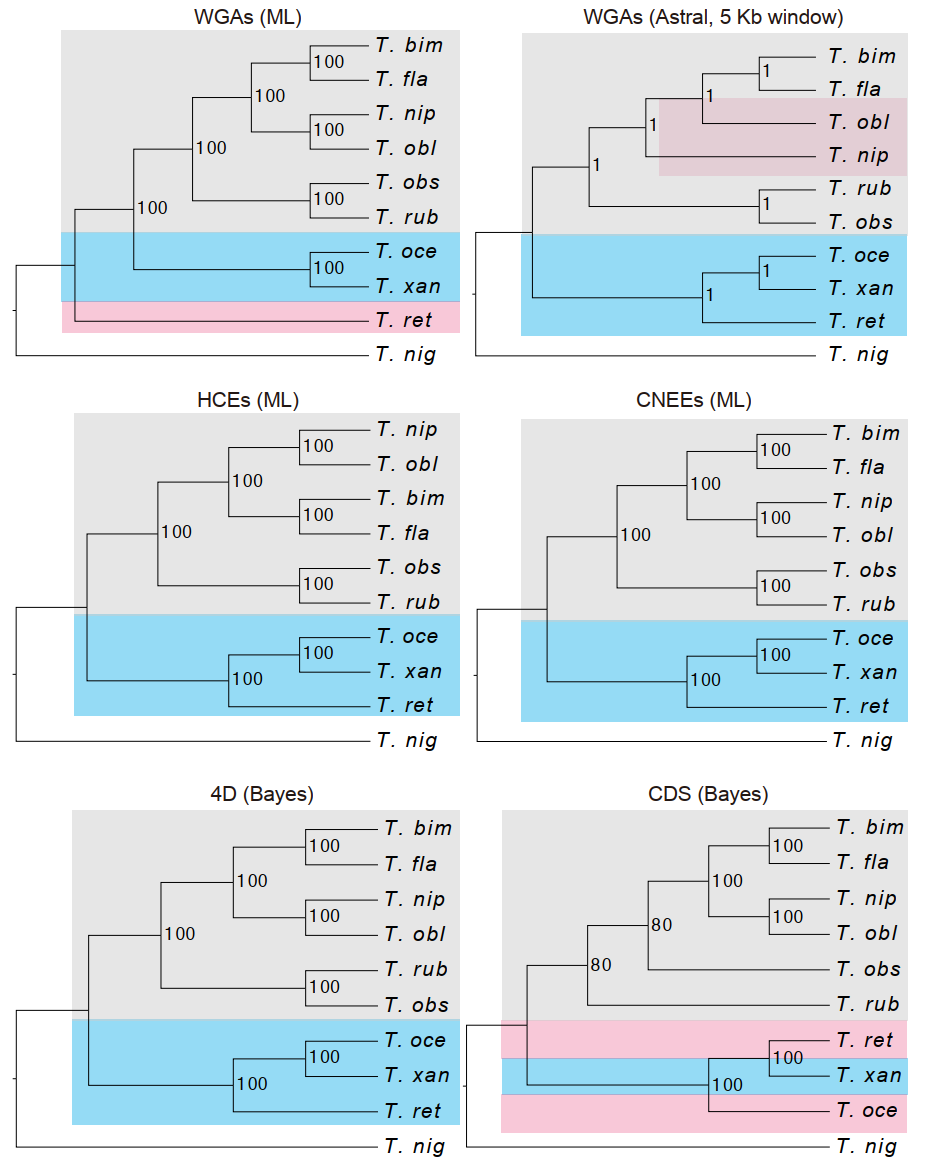
**

**Figure S9 | Phylogenetic tree construction by different approaches.** Different clade is shaded by grey and blue, while minor discrepancies are shaded in red. The topological relationship was reconstructed by Maximum likelihood (ML) method, Bayesian inference (Bayes) method and multispecies coalescent model (MSC, Astral).

**
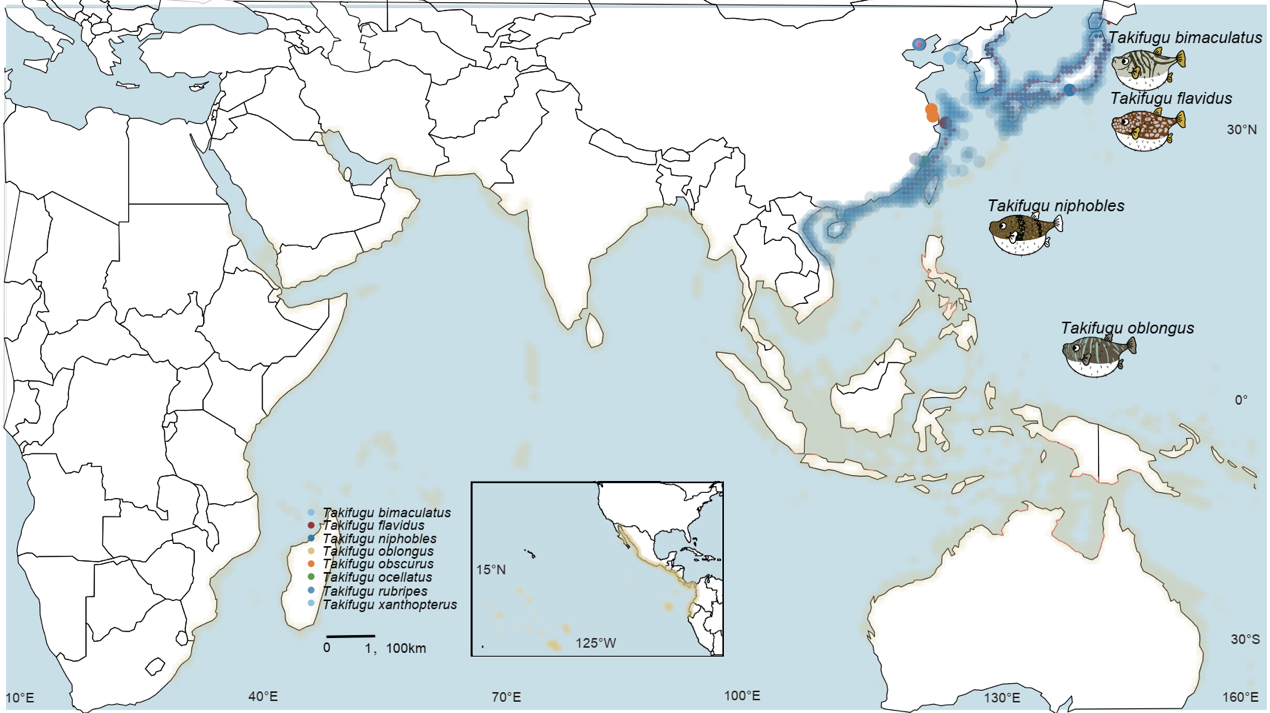
**

**Figure S10 | The geographic distribution of *Takifugu*,** with different colors representing the distribution ranges of various species. The geographical distribution of *T. reticularis* is not available.


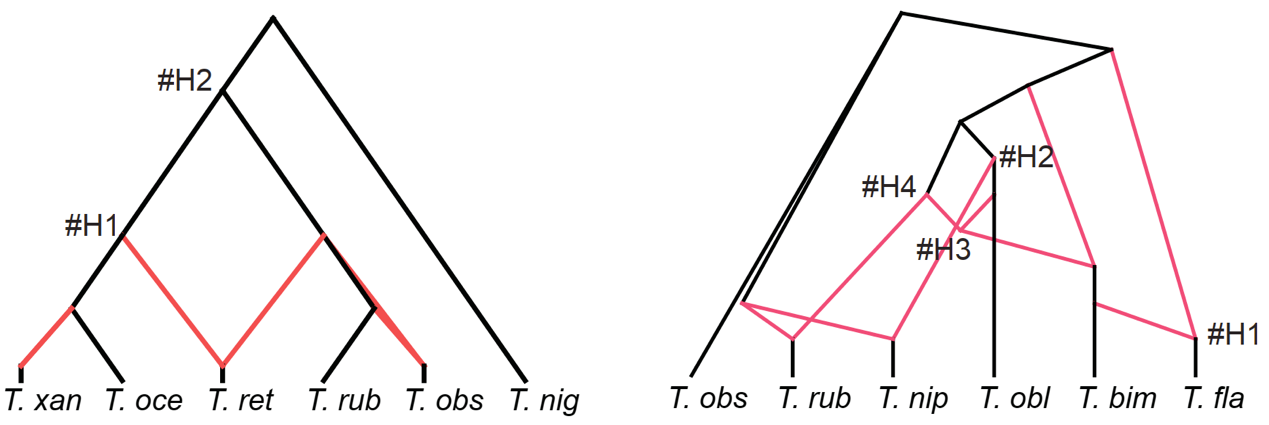


**Figure S11 | Phylogenetic networks using PhyloNet Infer_Network_MPL.** Left: *T. ret* clade network. Right: *T. obs* clade network. #H1, #H2, #H3, and #H4 denotes the hybridization events.


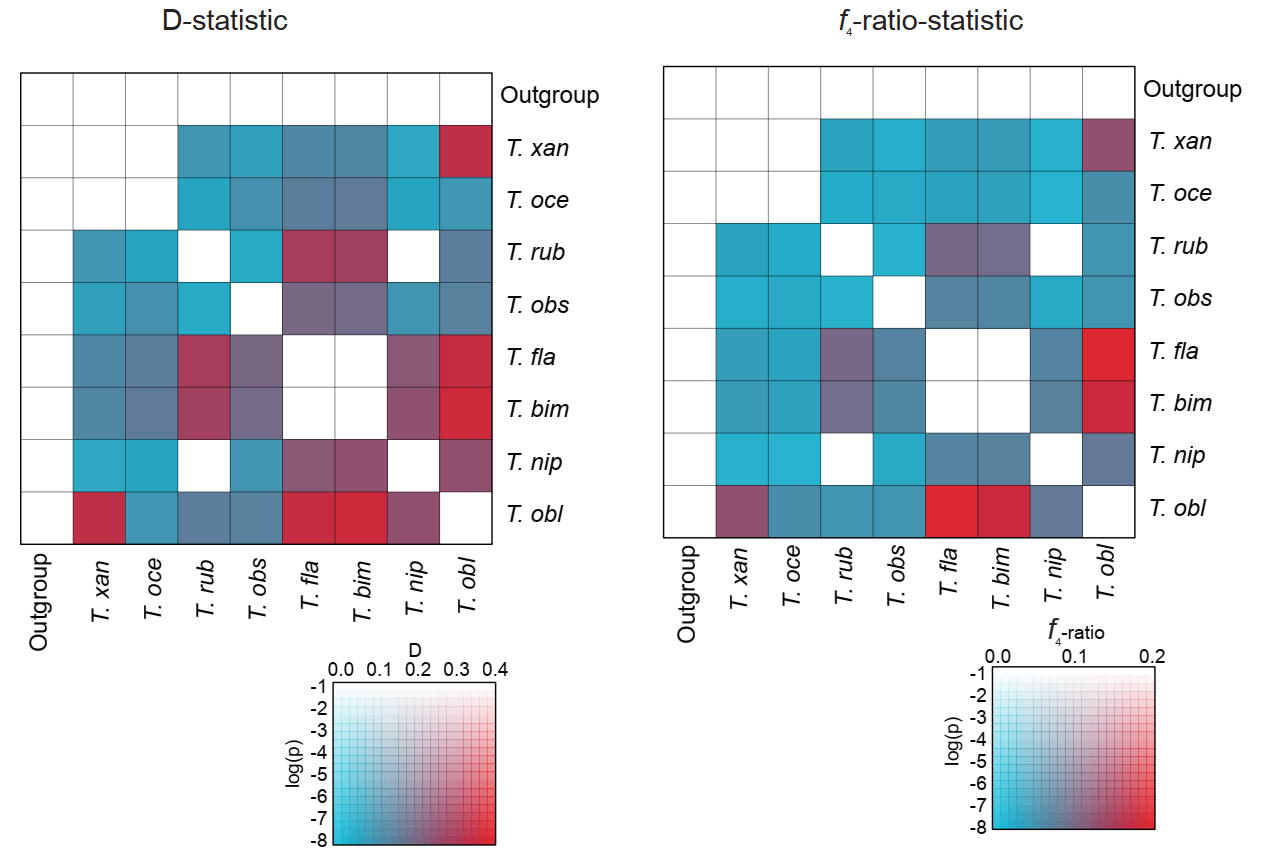


**Figure S12 | Results from *D*-statistics and *f*_4_-statistics showed the genetic affinity between nine pufferfish.** The red color showed the population showed a close relationship with the right populations compared to the bottom populations. Blue color with negative values showed a close genetic relationship with bottom populations compared with the right populations


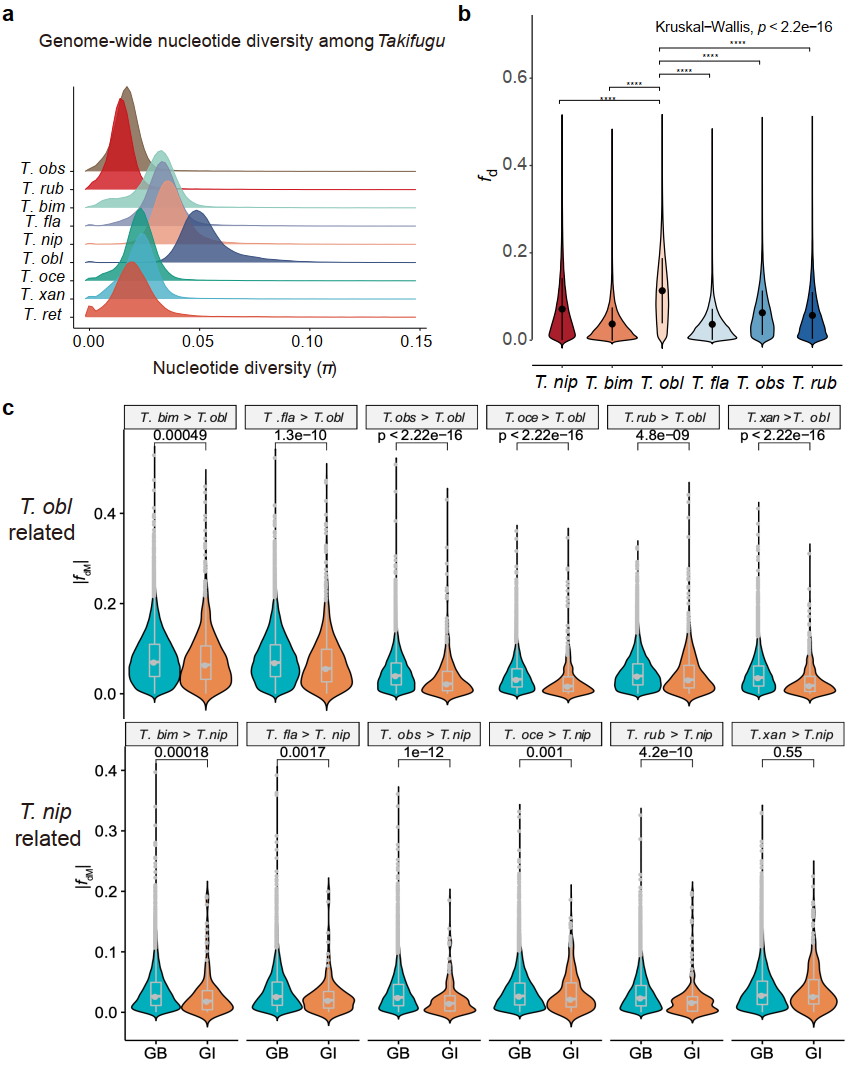


**Figure S13 | The evidence of the enhanced fitness of species *T. obl*.** **a,** Genome-wide nucleotide diversity among genus *Takifugu*. **b,** The introgression ratio of studied species. Kruskal-Walllis test is performed, and *p*-values lower than 2.2e-16 is denoted by ****. **c,** The introgression ratio (*f*_DM_) comparison between genomic islands (GI) and genomic background (GB) of close sister species *T. obl* and *T. nip*. Wilcoxon test is performed from package ggpubr (v0.4.0), and *p*-values lower than 0.05 was significantly different between GB and GI.


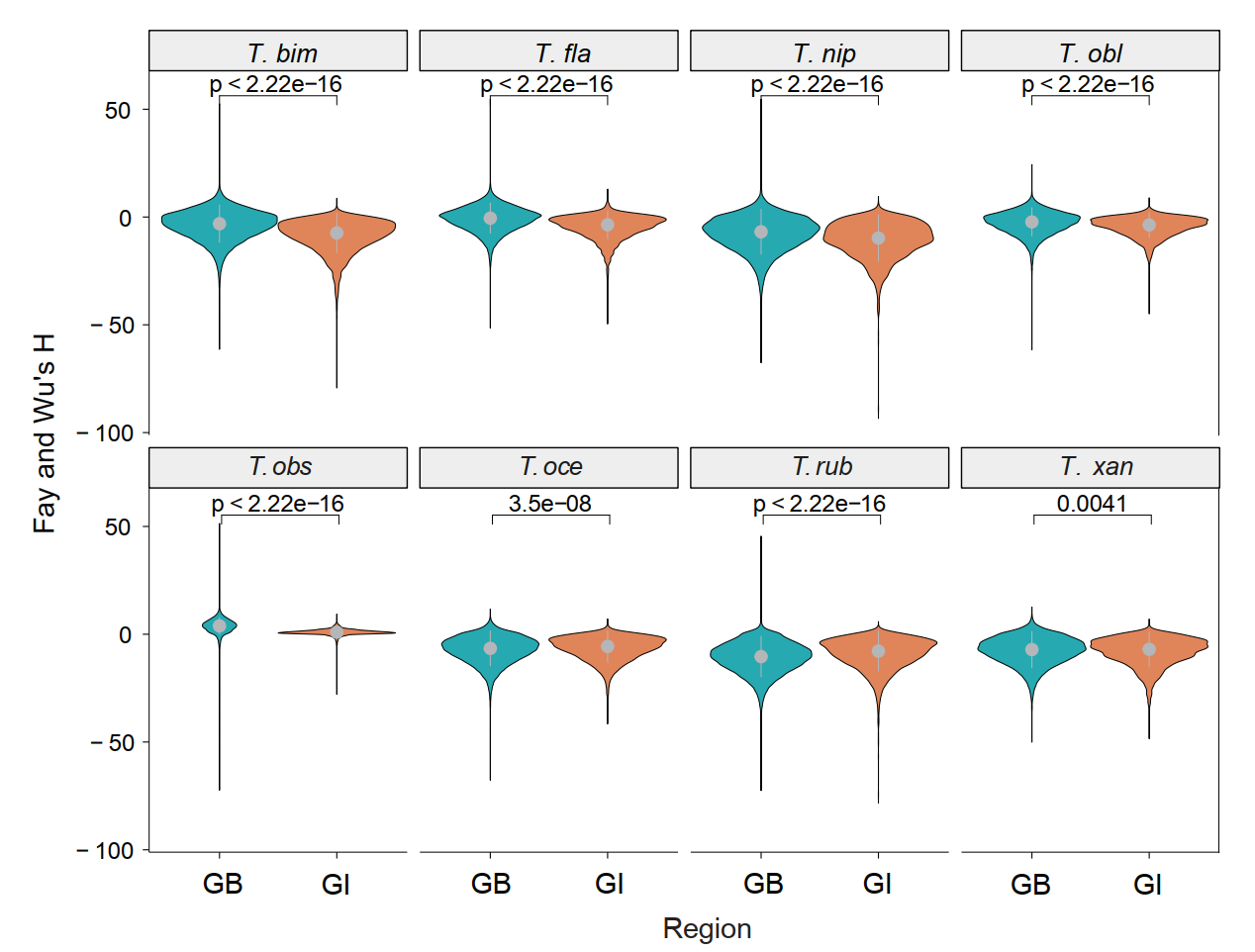


**Figure S14 | The Fay and Wu's H comparison between genomic islands (GI) and genomic background (GB) of *Takifugu* genome.** Wilcoxon test is performed, and *p*-values lower than 0.05 was significantly different between GB and GI.


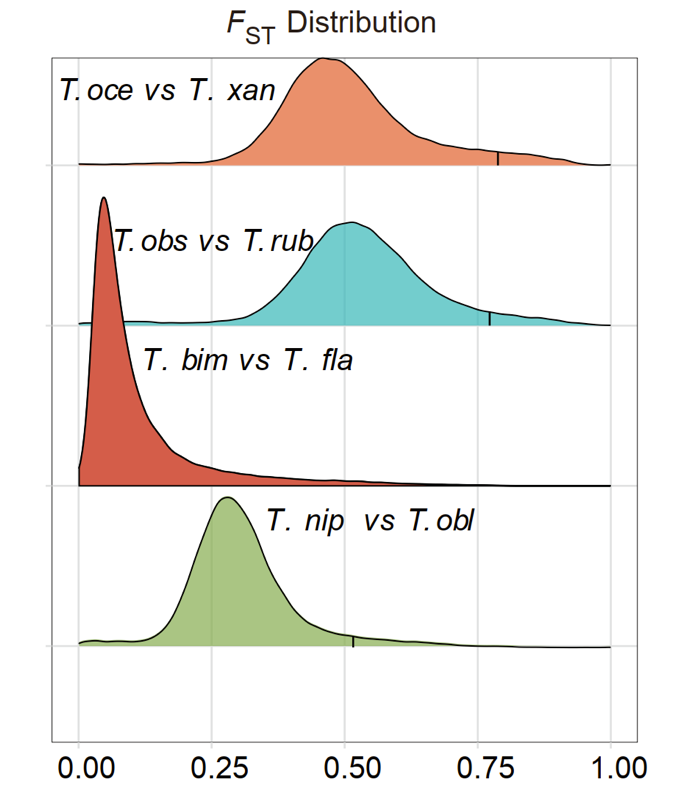


**Figure S15 | Ridge plot showing the fixation index (*F*_ST_) distribution of close sister species.**


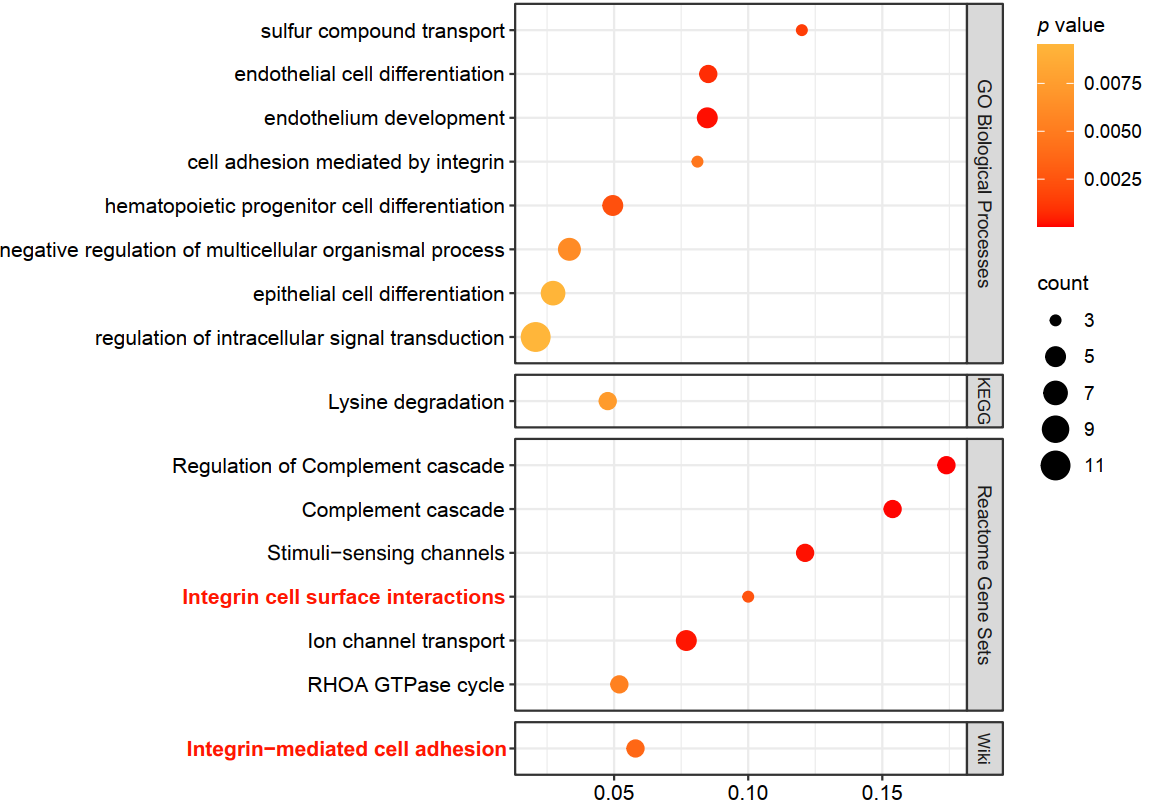


**Figure S16 | The circular bar plot showing the enrichment of positively selected genes in 12 fish** (10 pufferfish of this study, medaka and zebrafish) and *Takifugu* as foreground under the branch model.

**
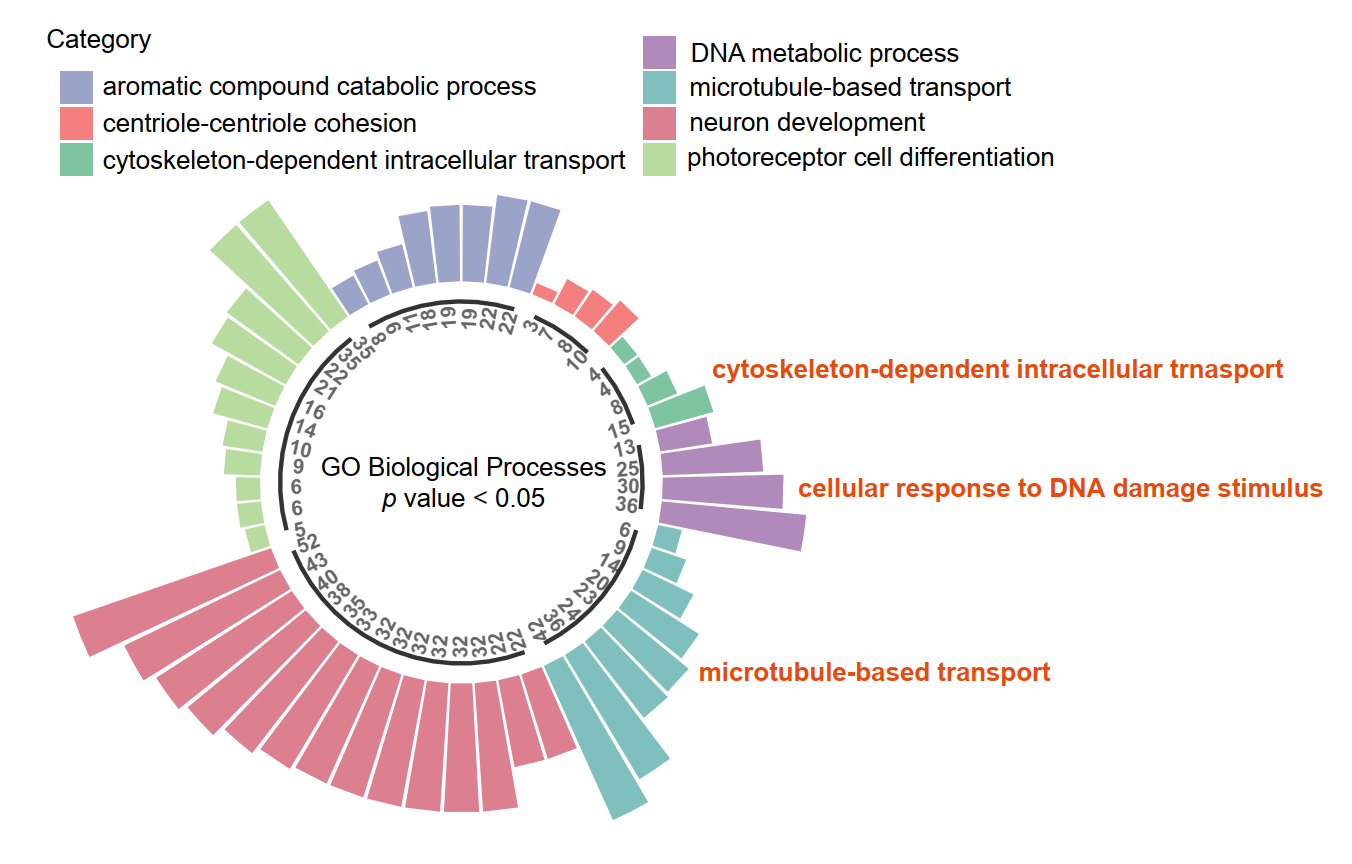
**

**Figure S17 | Plot of positive selection genes enrichment under branch model.** 10 pufferfish as foreground with 12 fish, 10 pufferfish of this study, medaka and zebrafish. Those genes are significant involved cytoskeleton-dependent intracellular transport, cellular response to DNA damage stimulus and microtubule-based transport.


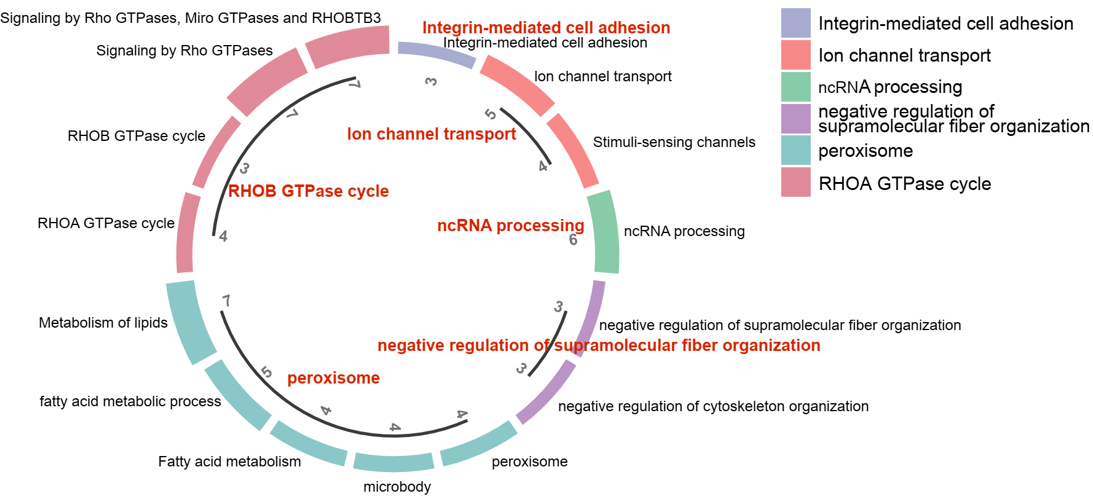


**Figure S18 | The barplot shows the enrichment of positively selected genes in 12 fish.** 10 pufferfish of this study, medaka and zebrafish, with *Takifugu* as foreground under the branch site model (removing the gene without positive selection sites).

**
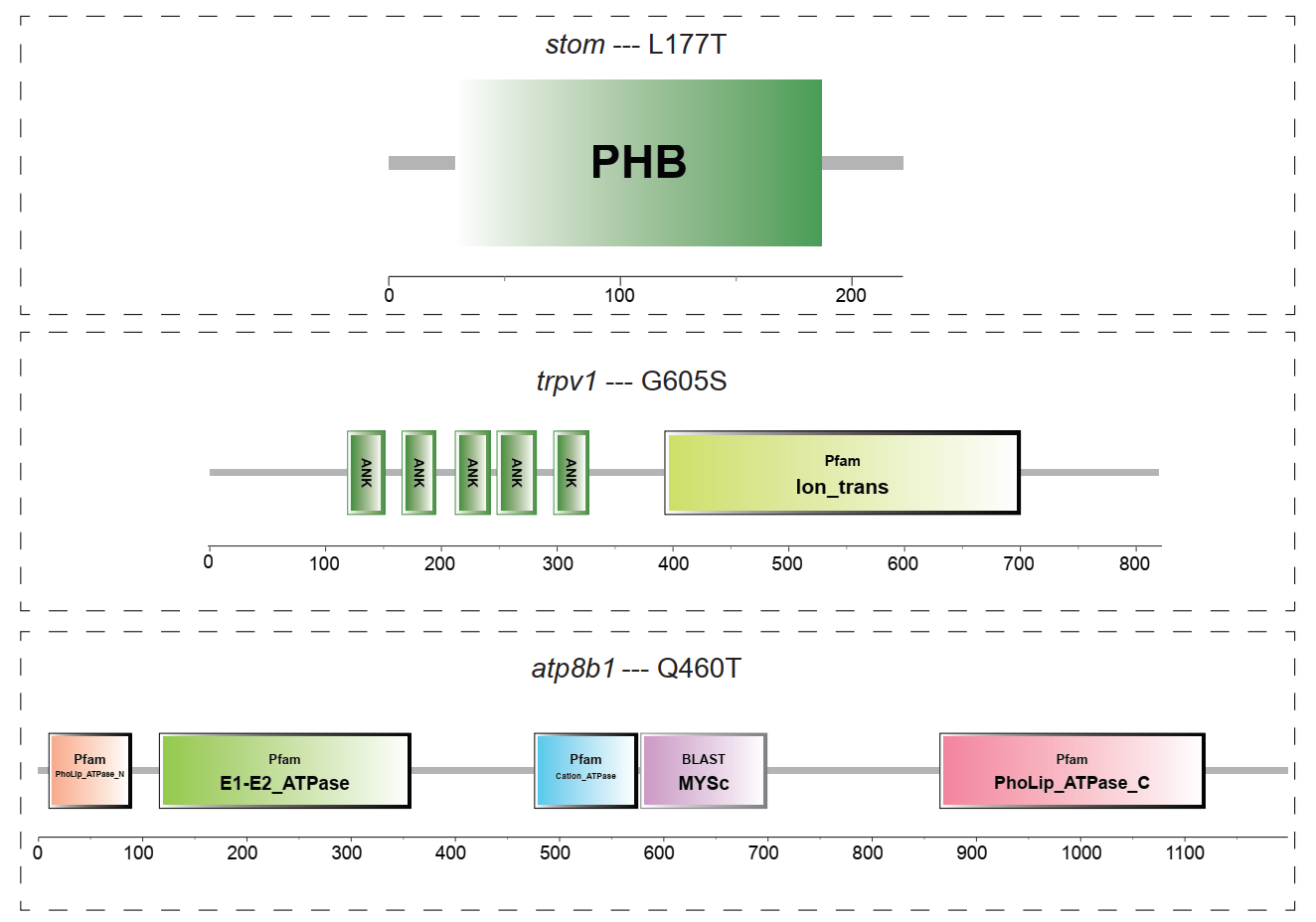
**

**Figure S19 | Diagram of the amino acid and domains of positive selection gene, *stom*, *trpv1*, *atp8b1*, among *Takifugu* compare to other fish.**


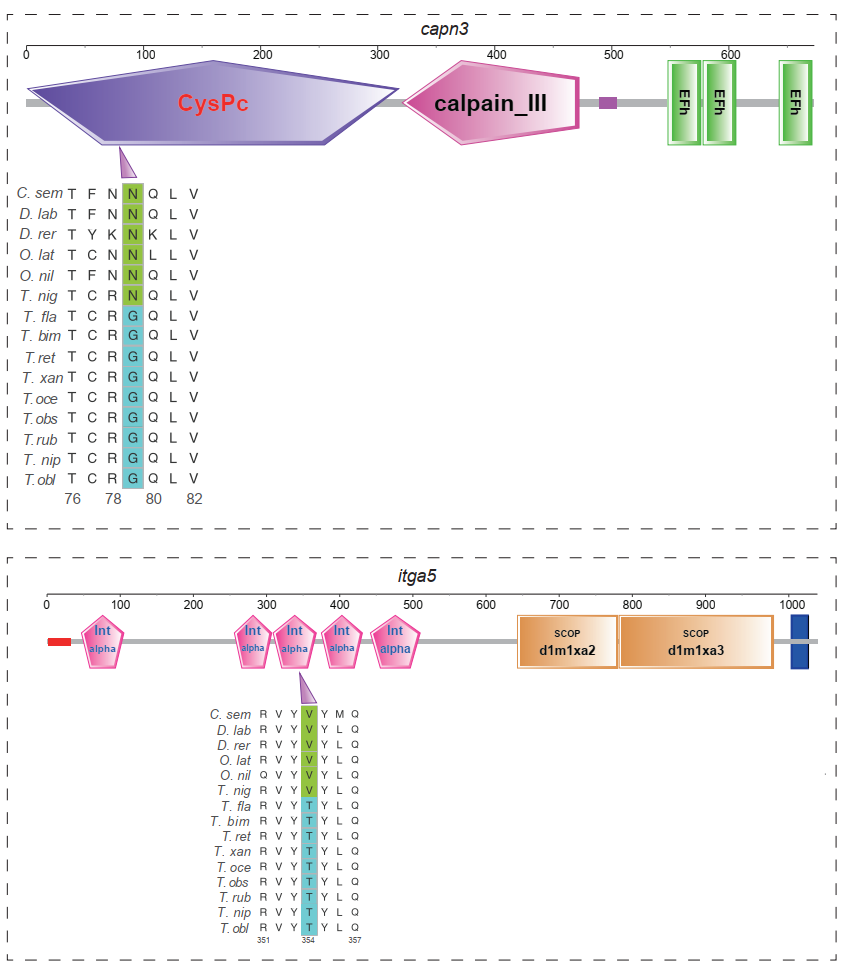


**Figure S20 | Diagram of the amino acid and domains of positive selection gene, *capn3* and *itga5*, among *Takifugu* compare to other fish.**


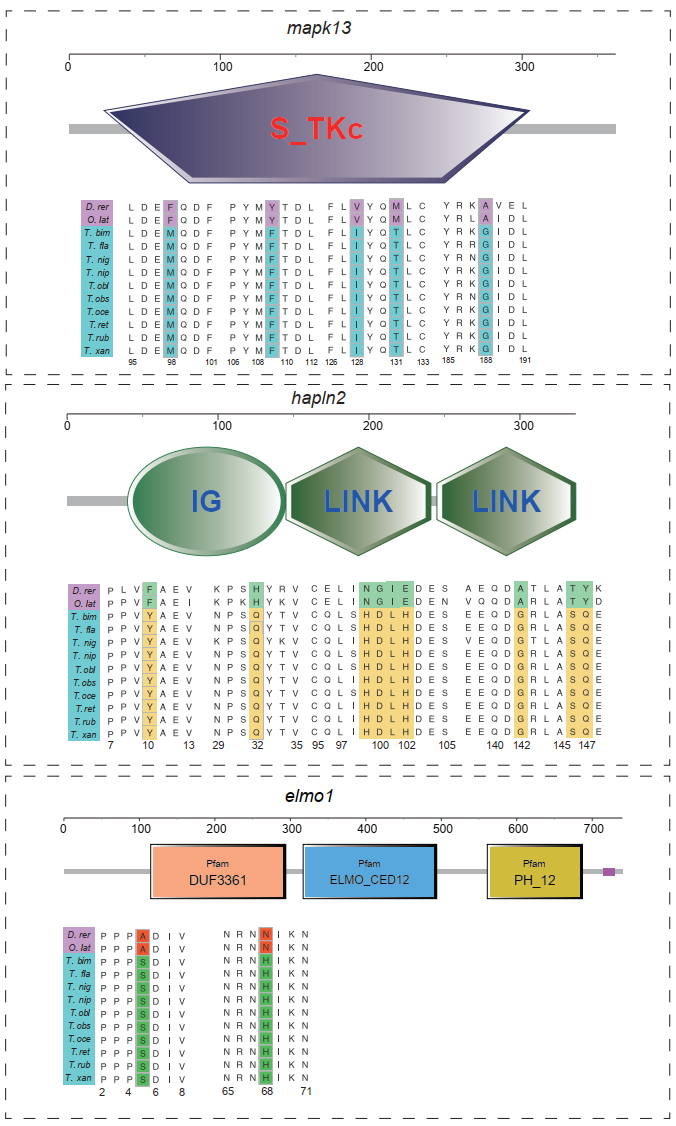


**Figure S21 | Diagram of the amino acid and domains of positive selection gene, *mapk13*, *hapln2* and *elmo1*, among pufferfish compare to other fish.**


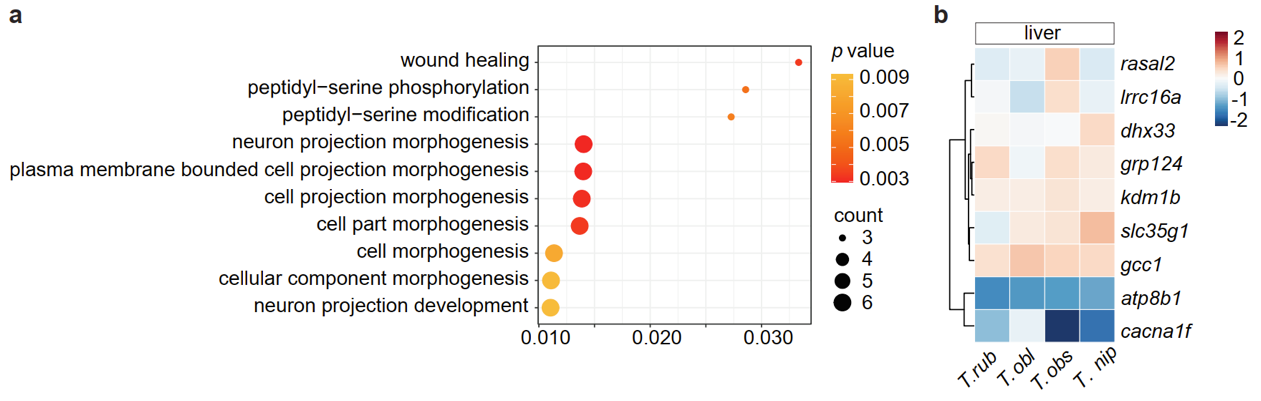


**Figure S22 | Genes within the genomic island of divergence among the genus *Takifugu*. a**, Enrichment of positively selected genes in genomic island of divergence. **b,** Expression of positively selected genes within genomic island of divergence in liver.


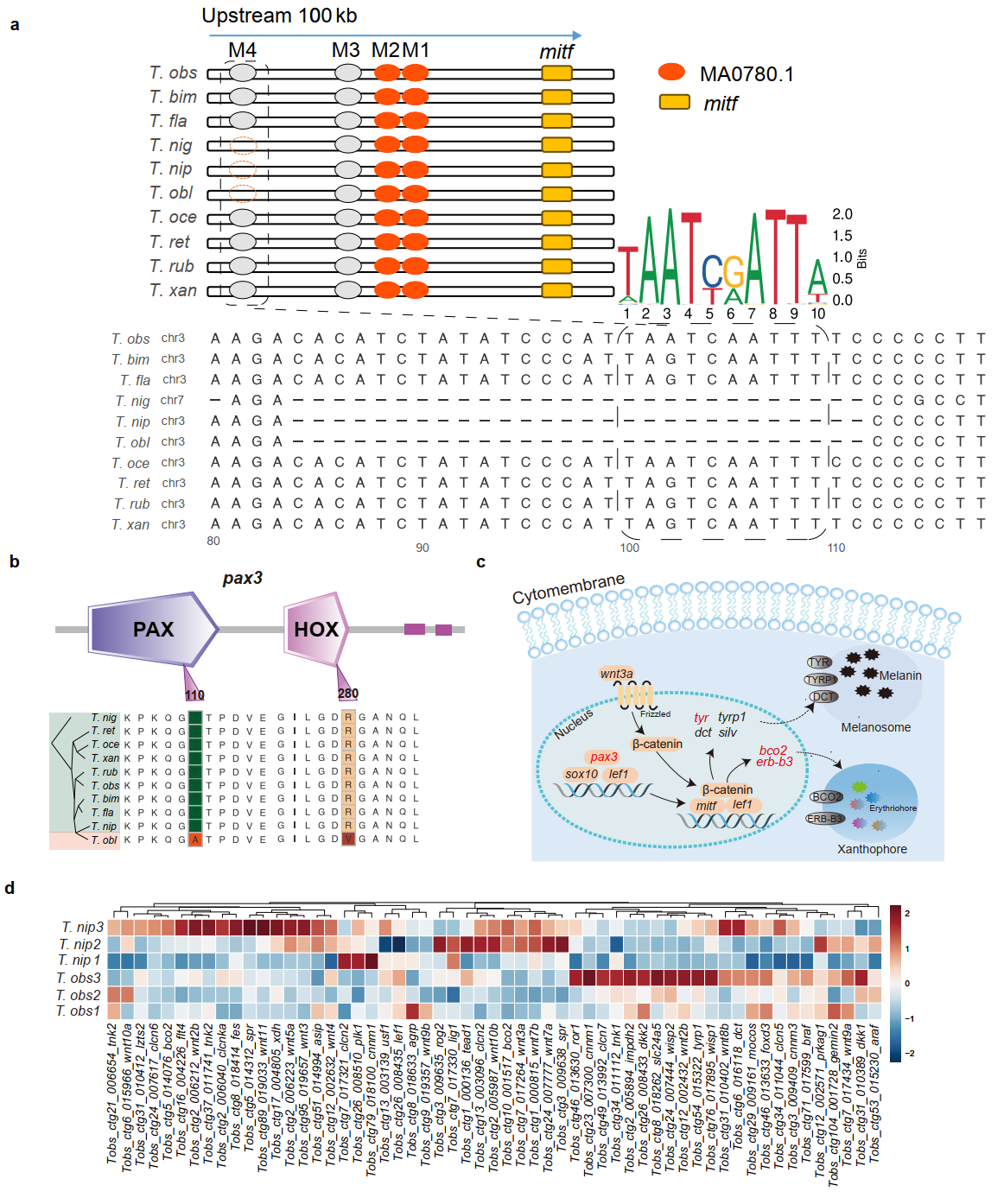


**Figure S23 | Key genes driving the divergence of color patterns in the skin of the genus *Takifugu*. a,** Diagram of *pax3* DNA binding site deletion among pufferfish. From the top panel, the red circle with shade is the core DNA motifs bound by PAX3 are present in the promoter region of gene *mitf* among pufferfish genome, while grey circle without shade is deletion of binding sites, the orange one is the gene *mitf*. The bottom panel showed the alignment of promoter region of *mitf* and corresponding motif sequence. **b,** Mutations of *pax3* in *T.obl* compared to other pufferfish. PAX and HOX represent the Paired-box and homeodomain, respectively. The two fixed substitutions (T110A, R280V) between *T. obl* and other pufferfish are highlighted by different color. **c,** Hypothetical signaling pathway that may correlate with color pattern differences among species of *Takifugu*. Genes marked with red are those have undergone positive selection in different *Takifugu* species*.* **d,** Heatmap showing skin coloration and pigmentation genes among species of the genus *Takifugu*.


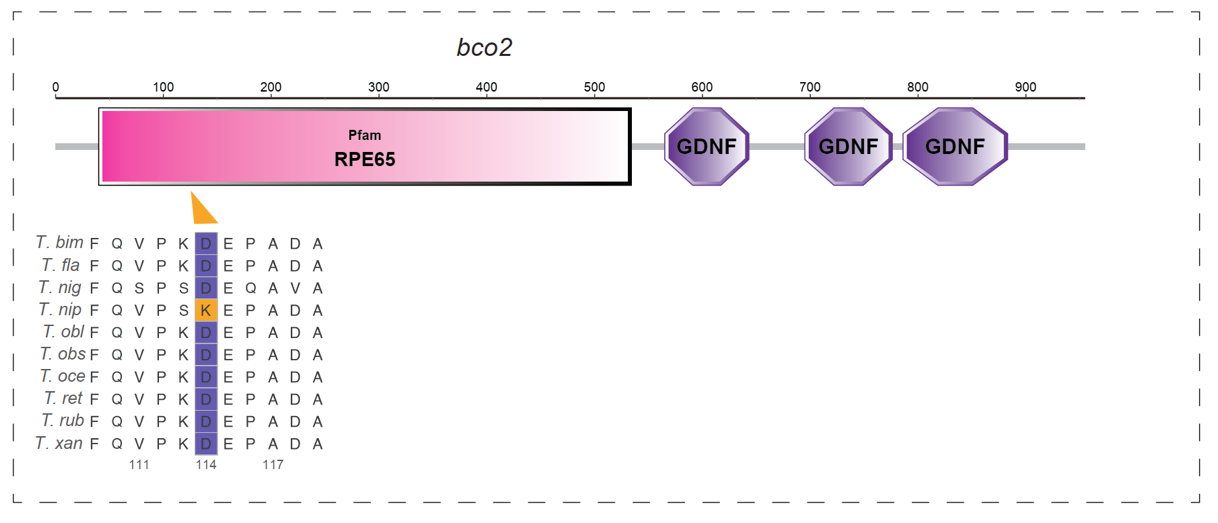


**Figure S24 | Diagram of the amino acid of positive selection gene *bco2* in *T. nip* compare to pufferfish.** The orange shade marked the changed amino acid.


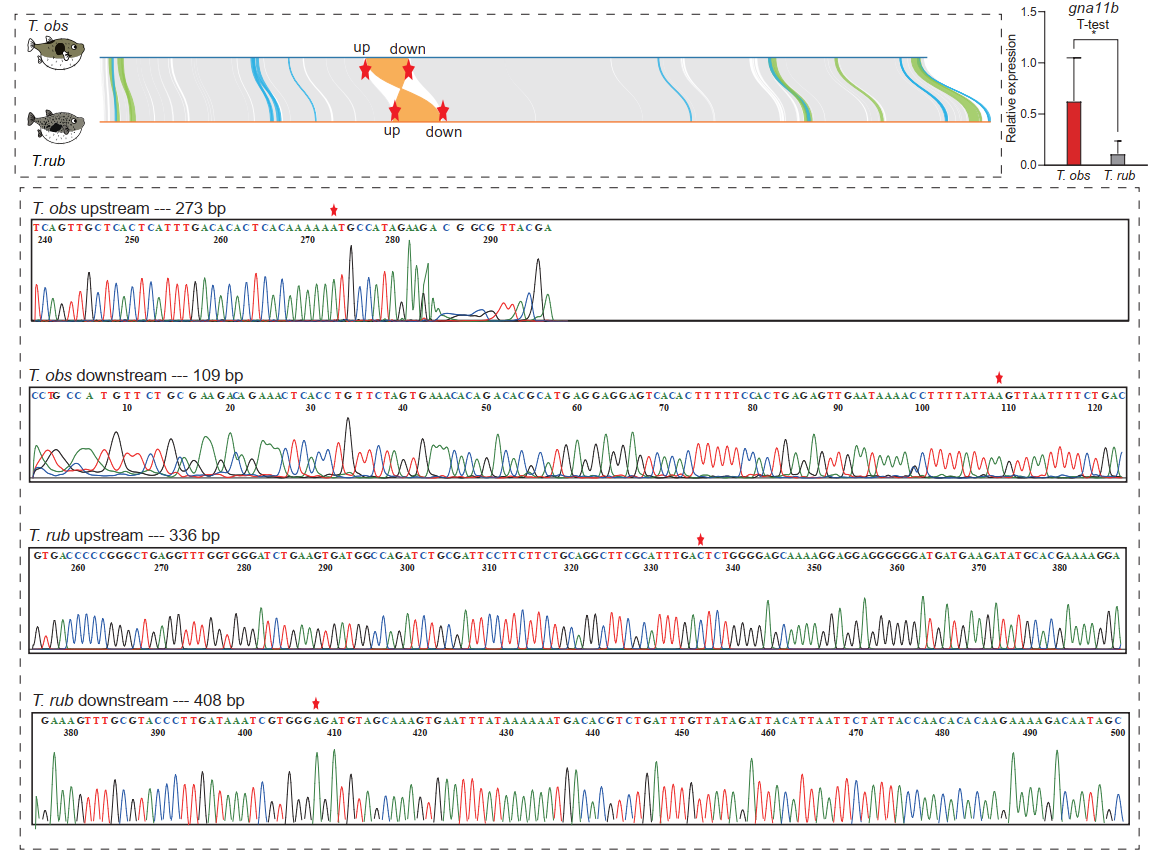


**Figure S25 | The Sanger-sequencing results of validated inversion between *T. obs* and *T. rub* and the gene expression of color-related gene.** The red star represents the inversion-caused breakpoint. The expression levels of gene *gna11b* associated with skin coloration. Expression levels were evaluated by RT-qPCR between two species. The T-test is conducted, and statistical significance is denoted as follows: **p* < 0.05.

# Supporting Information tables

**Table S1 | Fugu sample and data generated in this study.**

|  |  | |  | |  | |  | | |  |  |  |
| --- | --- | --- | --- | --- | --- | --- | --- | --- | --- | --- | --- | --- |
| Species | | Abbre. | | Long reads  (Gb)/Fold(X)^a^ | | Hi-C (Gb) | | NGS (Gb) | RNA-seq (Gb) | | Methylation tissue^b^ | ATAC (Gb)^c^ |
| *Takifugu oblongus* | | *T. obl* | | 29.58/84 | | 79.48 | | 36.48 | 35.57 | | liver | 18.11 (brain/heart) |
| *Takifugu niphobles* | | *T. nip* | | 29.40/84 | | 131.29 | | 58.99 | 31.66 | | liver/intestine | NA |
| *Takifugu flavidus* | | *T. fla* | | 29.98/86 | | 107.21 | | 37.80 | 14.48 | | NA | NA |
| *Takifugu bimaculatus* | | *T. bim* | | 44.90/128 | | 105.43 | | 45.12 | NA | | liver/intestine | NA |
| *Takifugu obscurus* | | *T. obs* | | 24.29/69 | | 312.26 (69.84 scaffolding) | | 17.11 | 61.12 | | liver/intestine | 34.39 (brain/liver/kidney) |
| *Takifugu rubripes* | | *T. rub* | | 15.16/43 | | 446.85 (131.44 scaffolding) | | 40.37 | 4.96 | | liver/intestine | NA |
| *Takifugu xanthopterus* | | *T. xan* | | 29.92/85 | | 144.92 | | 38.61 | 4.88 | | liver/intestine | NA |
| *Takifugu ocellatus* | | *T. oce* | | 49.4/141 | | 138.76 | | 37.53 | 24.89 | | liver/intestine | NA |
| *Takifugu reticularis* | | *T. ret* | | 27.43/78 | | 83.65 | | 44.51 | NA | | NA | NA |
| *Tetraodon nigroviridis* | | *T. nig* | | 15.97/45 | | 137.17 | | 36.47 | NA | | NA | NA |

^a^ We adopted the genome size of 350 Mb for these pufferfish.

^b^ The samples from *T. obs* and *T. rub* are adult individual, while the corresponding information of other fish (from wild) are not available.

^c^ The samples from *T. obs* are 2-year-old individuals, while the corresponding information from *T.* *obl* (from wild) is not available.

**Table S2 | Genome assembly and annotation statistics of studied fugu genome.**

|  | |  | |  | |  |  |  |  |  |  |  |  |  |
| --- | --- | --- | --- | --- | --- | --- | --- | --- | --- | --- | --- | --- | --- | --- |
| Species | Genome length (bp) (chromosome-level) | | Contig N50 (bp) | | Scaffold N50 (bp) | | Anchor ratio | Gene count | Total gene length (bp) | Average gene length (bp) | Total exon length (bp) | Total intron length (bp) | Average intron length(bp) | UTR ratio |
| *T. obl* | 371,954,429 | | 5,513,366 | | 16,062,624 | | 99.62% | 20,376 | 209,109,510 | 11,268 | 37,490,474 | 171,619,036 | 854 | 56.81% |
| *T. nip* | 385,029,667 | | 5,135,401 | | 16,765,194 | | 98.58% | 20,324 | 206,514,655 | 10,894 | 37,550,820 | 168,963,835 | 853 | 55.02% |
| *T. fla* | 373,078,169 | | 5,343,969 | | 16,821,030 | | 98.75% | 20,156 | 209,476,033 | 10,806 | 37,443,335 | 172,032,698 | 831 | 30.25% |
| *T. bim* | 372,680,988 | | 5,309,571 | | 16,699,104 | | 99.72% | 20,098 | 208,302,343 | 11,555 | 37,079,882 | 171,222,461 | 854 | 55.03% |
| *T. obs* | 360,631,485 | | 5,222,189 | | 16,040,510 | | 99.32% | 19,785 | 204,741,333 | 11,429 | 37,705,693 | 167,035,640 | 828 | 50.42% |
| *T. rub* | 362,635,475 | | 6,266,849 | | 16,027,113 | | 99.59% | 20,047 | 200,667,553 | 11,076 | 37,236,599 | 163,430,954 | 824 | 51.36% |
| *T. xan* | 356,459,658 | | 6,804,844 | | 15,932,125 | | 99.76% | 19,877 | 193,035,678 | 10,150 | 36,498,804 | 156,536,874 | 805 | 40.74% |
| *T. oce* | 355,693,761 | | 8,129,047 | | 16,038,496 | | 99.76% | 19,342 | 202,302,079 | 11,392 | 37,336,073 | 164,966,006 | 820 | 51.65% |
| *T. ret* | 381,030,615 | | 4,714,613 | | 16,962,205 | | 95.52% | 19,402 | 209,799,260 | 10,828 | 36,745,643 | 173,346,952 | 877 | NA |
| *T. nig* | 346,484,523 | | 8,150,701 | | 14,221,180 | | 90.03% | 19,050 | 163,272,100 | 8,571 | 34,700,271 | 128,571,829 | 706 | 48.68% |

**Table S3 | TAD distribution and variation across chromosomes.**

|  | | | | | |  |
| --- | --- | --- | --- | --- | --- | --- |
| Species | Tissue | TAD count | Total TAD length  (bp) | Average TAD length (bp) | Genome length  (bp) | TAD genome  ratio |
| *T. obl* | liver | 513 | 298,015,000 | 580,926 | 371,954,429 | 80.12% |
| *T. nip* | liver | 621 | 309,452,500 | 498,313 | 385,029,667 | 80.37% |
| *T. fla* | blood | 708 | 329,900,000 | 465,960 | 373,078,169 | 88.43% |
| *T. bim* | blood | 581 | 308,285,000 | 530,611 | 372,680,988 | 82.72% |
| *T. bim* | muscle | 592 | 312,820,000 | 528,412 | 372,680,988 | 83.94% |
| *T. obs* | blood | 719 | 309,415,000 | 430,341 | 360,631,485 | 85.80% |
| *T. obs* | muscle | 642 | 319,460,000 | 497,601 | 360,631,485 | 88.58% |
| *T. obs* | skin | 703 | 322,780,000 | 459,147 | 360,631,485 | 89.50% |
| *T. rub* | blood | 631 | 309,237,500 | 490,075 | 362,635,475 | 85.28% |
| *T. rub* | muscle | 700 | 318,960,000 | 455,657 | 362,635,475 | 87.96% |
| *T. rub* | skin | 439 | 299,920,000 | 683,189 | 362,635,475 | 82.71% |
| *T. xan* | liver | 454 | 283542500 | 624,543 | 356,459,658 | 79.54% |
| *T. oce* | liver | 804 | 308,240,000 | 383,383 | 355,693,761 | 86.66% |
| *T. ret* | liver | 634 | 318,720,000 | 502,713 | 381,030,615 | 83.65% |

**Table S4 | Reference-based TAD comparison of same tissue in different species.**

| Species | Tissue | Original TAD count | Shared TAD count | Shared ratio |
| --- | --- | --- | --- | --- |
| *T. obs (ref)* | skin | 725 | 236 | 32.55% |
| *T. rub* | skin | 440 | 237 | 53.86% |
| Average shared |  |  |  | 43.21% |
| *T. obs* | muscle | 660 | 461 | 69.85% |
| *T. bim* | muscle | 575 | 256 | 44.52% |
| *T. rub* | muscle | 688 | 342 | 49.71% |
| Average shared |  |  |  | 54.69% |
| *T. bim* | blood | 573 | 286 | 49.91% |
| *T. fla* | blood | 692 | 347 | 50.14% |
| *T. rub* | blood | 627 | 334 | 53.27% |
| *T. obs* | blood | 727 | 578 | 79.50% |
| Average shared |  |  |  | 58.21% |
| *T. nip* | liver | 597 | 295 | 49.41% |
| *T. obl* | liver | 500 | 273 | 54.60% |
| *T. oce* | liver | 776 | 612 | 78.87% |
| *T. ret* | liver | 612 | 298 | 48.69% |
| *T. xan* | liver | 459 | 221 | 48.15% |
| Average shared |  |  |  | 55.94% |
| All average shared |  |  |  | 54.50% |

**Table S5 | Repeat annotation summary of studied fugu genome.**

| Species | DNA transposons (%) | LINEs (%) | SINEs (%) | LTRs (%) | Unknown (%) | Total (%) |
| --- | --- | --- | --- | --- | --- | --- |
| *T. bim* | 8.38 | 9.39 | 0.39 | 6.86 | 0.23 | 19.70 |
| *T. obl* | 8.33 | 8.72 | 0.34 | 6.55 | 0.18 | 18.82 |
| *T. obs* | 7.38 | 8.95 | 0.37 | 6.15 | 0.26 | 17.26 |
| *T. oce* | 7.44 | 8.70 | 0.36 | 4.97 | 0.52 | 16.12 |
| *T. ret* | 8.10 | 10.24 | 0.41 | 7.39 | 0.20 | 19.92 |
| *T. xan* | 7.07 | 8.03 | 0.34 | 5.64 | 0.25 | 15.88 |
| *T. nip* | 9.77 | 9.60 | 0.37 | 6.64 | 0.09 | 21.10 |
| *T, rub* | 7.07 | 8.60 | 0.35 | 5.71 | 0.13 | 16.97 |
| *T. fla* | 5.84 | 5.08 | 0.28 | 4.25 | 1.77 | 15.48 |
| *T. nig* | 11.31 | 11.17 | 0.38 | 5.04 | 0.52 | 22.59 |
| Average | 8.07 | 8.85 | 0.36 | 5.92 | 0.42 | 18.39 |

**Table S6 | The gene structure comparison among 74 vertebrate genomes of this study.**

See the Table S6 in the format of Excel.

**Table S7 | The statistics of whole genome alignment, highly conserved elements, and conserved non-exonic elements of this study.**

|  | Chr | Whole genome alignment (WGA) | Highly conserved elements (HCEs) | Conserved non-exonic elements (CNEEs) |
| --- | --- | --- | --- | --- |
| chromosome | Length(bp) | Length (bp) | Length (bp) | Length (bp) |
| chr1 | 29,106,024 | 27,872,623 | 8,402,752 | 5,891,083 |
| chr2 | 14,570,390 | 13,762,307 | 4,378,088 | 3,017,250 |
| chr3 | 17,137,321 | 16,083,563 | 5,172,972 | 3,638,913 |
| chr4 | 16,063,458 | 15,371,869 | 4,856,752 | 3,694,350 |
| chr5 | 14,486,874 | 12,994,241 | 3,813,729 | 2,465,794 |
| chr6 | 12,571,497 | 12,146,260 | 3,577,131 | 2,414,514 |
| chr7 | 16,234,534 | 15,816,170 | 5,151,734 | 3,811,591 |
| chr8 | 19,302,979 | 18,186,943 | 4,499,873 | 3,127,020 |
| chr9 | 15,714,919 | 14,970,779 | 4,926,953 | 3,387,790 |
| chr10 | 13,672,710 | 12,305,773 | 3,550,043 | 2,704,149 |
| chr11 | 15,942,374 | 15,049,745 | 4,110,746 | 2,923,498 |
| chr12 | 12,548,393 | 12,116,152 | 3,195,365 | 2,212,550 |
| chr13 | 19,742,640 | 19,174,067 | 5,835,524 | 4,500,499 |
| chr14 | 16,040,510 | 15,085,926 | 4,731,085 | 3,721,687 |
| chr15 | 15,635,825 | 14,319,930 | 3,857,423 | 2,609,945 |
| chr16 | 12,770,419 | 11,923,690 | 3,393,243 | 2,279,831 |
| chr17 | 15,985,689 | 15,258,855 | 4,265,591 | 2,811,560 |
| chr18 | 10,558,790 | 9,878,861 | 2,608,461 | 1,841,789 |
| chr19 | 19,115,316 | 16,548,111 | 4,641,612 | 3,232,319 |
| chr20 | 17,096,804 | 16,108,845 | 4,931,085 | 3,573,074 |
| chr21 | 18,573,316 | 17,265,029 | 5,263,377 | 3,728,027 |
| chr22 | 15,293,929 | 14,554,327 | 4,175,243 | 2,798,047 |
| Total | 358,164,711 | 336,794,066 | 99,338,782 | 70,385,280 |

Note: This set includes *T. obs, T. rub, T. fla, T. oce, T. ret, T. xan, T. bim, T. nip, T. obl, T. nig, O. lat,*and *D. rer,*

**Table S8 | Methods for phylogenetic framework of this study.**

| Strategy | Dataset | Sites | Taxonomy | Methods |
| --- | --- | --- | --- | --- |
| Concatenation | Whole genome alignments (WGAs) | 336,624,334 | 10 | Maximum likelihood (ML) |
|  | Highly conserved elements (HCEs) | 88,634,776 | 10 | Maximum likelihood (ML) |
|  | Conserved non-exonic elements (CNEEs) | 42,341,088 | 10 | Maximum likelihood (ML) |
|  | Protein-coding sequences (CDS) | 28,252,554 | 10 | Maximum likelihood (ML) |
|  | Protein-coding sequences (CDS) | 28,252,554 | 10 | Bayesian inference (Bayes) |
|  | Four-fold sites (4D) | 2,487,619 | 10 | Maximum likelihood (ML) |
|  | Four-fold sites (4D) | 2,487,619 | 10 | Bayesian inference (Bayes) |
| Multi-species coalescent (MSC) | WGAs 5k window | 336,624,334 | 10 | Maximum likelihood (ML) + Accurate  Species TRee Algorithm (Astral) |
|  | WGAs 10k window | 336,624,334 | 10 | Maximum likelihood (ML) + Accurate  Species TRee Algorithm (Astral) |
|  | WGAs 50k window | 336,624,334 | 10 | Maximum likelihood (ML) + Accurate  Species TRee Algorithm (Astral) |
|  | CNEEs 50k window | 42,341,088 | 10 | Maximum likelihood (ML) + Accurate  Species TRee Algorithm (Astral) |
|  | CNEEs 50k window | 42,341,088 | 10 | Maximum likelihood (ML) + Maximum  pseudo-likelihood estimation of species trees (MP-EST) |

**Table S9 | The result of QulBL analysis of this study.**

See the Table S9 in the format of Excel.

**Table S10 | Re-sequencing sample used in this study.**

See the Table S10 in the format of Excel.

**Table S11 | The summary statistics of SNP calling of this study**

| **Item** | **Count** |
| --- | --- |
| number of samples | 230 |
| number of records | 28,049,384 |
| number of no-ALTs | 0 |
| number of SNPs | 28,049,384 |
| number of multiallelic sites | 3,103,519 |
| number of multiallelic SNP sites | 3,103,519 |
| number of SNPs of final SNPs set | 24,945,865 |

**Table S12 | The mapping summary statistics of this study.**

See the Table S12 in the format of Excel.

**Table S13 | The result of Dsuite analysis of this study.**

See the Table S13 in the format of Excel.

**Table S14 | The result of Hyde analysis of this study.**

See the Table S14 in the format of Excel.

**Table S15 | The result of enrichment analysis of positive selection genes under the branch model.**

See the Table S15 in the format of Excel.

**Table S16 | ATAC-seq Peak statistics of *Takifugu obscurus.***

| Tissue | Peak count | Total peak length (bp) | Average length (bp) |
| --- | --- | --- | --- |
| Liver | 31,377 | 19,306,704 | 615.3139 |
| Brain | 30,209 | 18,033,137 | 596.9458 |
| Kidney | 30,039 | 14,048,760 | 467.684 |
| Combined | 58,308 | 34,158,907 | 585.8357 |

**Table S17 | Genome-wide pairwise differentiation *F*_ST_ (mean genetic differentiation) and Dxy (absolute genetic divergence) of the genus *Takifugu.***

| **Species 1** | **Species 2** | **Mean *F*_ST_** | **Mean Dxy** |
| --- | --- | --- | --- |
| *T. bim* | *T. fla* | 0.110349 | 0.0477199 |
| *T. bim* | *T. nip* | 0.364087 | 0.0993231 |
| *T. fla* | *T. nip* | 0.347079 | 0.0978019 |
| *T. bim* | *T. obl* | 0.264241 | 0.0909397 |
| *T. fla* | *T. obl* | 0.241147 | 0.0897439 |
| *T. nip* | *T. obl* | 0.256949 | 0.112318 |
| *T. bim* | *T. obs* | 0.433916 | 0.0765962 |
| *T. fla* | *T. obs* | 0.404687 | 0.0743319 |
| *T. nip* | *T. obs* | 0.48474 | 0.100212 |
| *T. obl* | *T. obs* | 0.345564 | 0.095944 |
| *T. bim* | *T. oce* | 0.483596 | 0.102674 |
| *T. fla* | *T. oce* | 0.458239 | 0.100919 |
| *T. nip* | *T. oce* | 0.423336 | 0.118765 |
| *T. obl* | *T. oce* | 0.37852 | 0.103814 |
| *T. obs* | *T. oce* | 0.533647 | 0.0986783 |
| *T. bim* | *T. ret* | 0.197243 | 0.109265 |
| *T. fla* | *T. ret* | 0.16674 | 0.107313 |
| *T. nip* | *T. ret* | 0.105313 | 0.124911 |
| *T. obl* | *T. ret* | 0.204447 | 0.124182 |
| *T. obs* | *T. ret* | 0.183529 | 0.103667 |
| *T. oce* | *T. ret* | 0.340689 | 0.125048 |
| *T. bim* | *T. rub* | 0.391143 | 0.078903 |
| *T. fla* | *T. rub* | 0.36715 | 0.0764684 |
| *T. nip* | *T. rub* | 0.492908 | 0.102259 |
| *T. obl* | *T. rub* | 0.275124 | 0.0978255 |
| *T. obs* | *T. rub* | 0.519103 | 0.0723425 |
| *T. oce* | *T. rub* | 0.442978 | 0.101017 |
| *T. ret* | *T. rub* | 0.104807 | 0.103637 |
| *T. bim* | *T. xan* | 0.413326 | 0.0882378 |
| *T. fla* | *T. xan* | 0.38223 | 0.0863943 |
| *T. nip* | *T. xan* | 0.347493 | 0.105674 |
| *T. obl* | *T. xan* | 0.331099 | 0.0934417 |
| *T. obs* | *T. xan* | 0.453602 | 0.0841554 |
| *T. oce* | *T. xan* | 0.497809 | 0.0882994 |
| *T. ret* | *T. xan* | 0.362111 | 0.110775 |
| *T. rub* | *T. xan* | 0.351236 | 0.0857064 |

**Table S18 | Nucleotide diversity (π) statistics of genus *Takifugu***

| **Species** | ***T. obl*** | ***T. nip*** | ***T. fla*** | ***T. bim*** | ***T. oce*** | ***T. xan*** | ***T. ret*** | ***T. obs*** | ***T. rub*** |
| --- | --- | --- | --- | --- | --- | --- | --- | --- | --- |
| **Mean (π)** | 0.064946 | 0.046234 | 0.040237 | 0.036960 | 0.028052 | 0.027907 | 0.027599 | 0.020842 | 0.018256 |
| **STDDEV** | 0.018 | 0.015 | 0.014 | 0.016 | 0.013 | 0.013 | 0.013 | 0.012 | 0.029 |

**Table S19 | The mutation rate estimation of genus *Takifugu***

| species | count | median | mean | maximum | minimum | stdev | variance | mutation rate |
| --- | --- | --- | --- | --- | --- | --- | --- | --- |
| *C.har※* | 6289 | 2.98 | 3.13 | 7.31 | 0.29 | 1.24 | 1.54 | 2.00E-09 |
| *T.bim* | 6289 | 4.02 | 3.67 | 8.01 | 0.41 | 1.16 | 1.34 | 2.70E-09 |
| *T.fla* | 6289 | 4 | 3.65 | 6.97 | 0.41 | 1.14 | 1.3 | 2.68E-09 |
| *T.nig* | 6289 | 4.06 | 3.74 | 8.65 | 0.41 | 1.11 | 1.23 | 2.72E-09 |
| *T.nip* | 6289 | 4.03 | 3.68 | 7.91 | 0.47 | 1.14 | 1.31 | 2.70E-09 |
| *T.obl* | 6289 | 4.03 | 3.69 | 8.38 | 0.47 | 1.15 | 1.32 | 2.70E-09 |
| *T.obs* | 6289 | 4.01 | 3.66 | 8.13 | 0.27 | 1.16 | 1.34 | 2.69E-09 |
| *T.oce* | 6289 | 4.02 | 3.68 | 11.73 | 0.47 | 1.15 | 1.33 | 2.70E-09 |
| *T.ret* | 6289 | 4.02 | 3.68 | 10.18 | 0.39 | 1.15 | 1.33 | 2.70E-09 |
| *T.rub* | 6289 | 4.01 | 3.66 | 7.73 | 0.47 | 1.15 | 1.31 | 2.69E-09 |
| *T.xan* | 6289 | 4 | 3.66 | 7.62 | 0.44 | 1.15 | 1.32 | 2.68E-09 |
| ※ Feng C, Pettersson M, Lamichhaney S, et al. Moderate nucleotide diversity in the *Atlantic herring* is associated with a low mutation rate. elife. 2017;6:e23907. doi:10.7554/eLife.23907 | | | | | | | | |

**Table S20 | Primers used in motif activity analysis.**

| Primer Name | Sequence (5’-3’) |
| --- | --- |
| M1+M2-*luc*-F | gcgtgctagcccgggctcgagTCAGCGAAGCACCTTCGAA |
| M1+M2-*luc*-R | cagtaccggaatgccaagcttTTCTCCGCTGGATCACTGTGA |
| M3-*luc*-F | gcgtgctagcccgggctcgagTATTCTGGGTCACCTCAGCTCT |
| M3-*luc*-R | cagtaccggaatgccaagcttTCATTGTGTTGCTTGATTTTCATG |
| M4-*luc*-F | ccgctcgagGGCACCTTTGGTTCCTATAA |
| M4-*luc*-R | ccaagcttCCCCTCTCAGACTTACCTTA |
| *pax3*-F | ctagcgtttaaacttaagcttATGTTGGACTTGTATCCATCCCC |
| *pax3*-R | tgctggatatctgcagaattcTACCTTGCTCTGACTGTACTGGCC |

Note: Sequence in lowercase is the restriction enzyme cutting site or homologous sequence of the vector.
